# A universal function for capacity of bidirectional pedestrian streams: Filling the gaps in the literature

**DOI:** 10.1371/journal.pone.0208496

**Published:** 2018-12-19

**Authors:** Claudio Feliciani, Hisashi Murakami, Katsuhiro Nishinari

**Affiliations:** 1 Department of Advanced Interdisciplinary Studies, Graduate School of Engineering, The University of Tokyo, Meguro-ku, Tokyo, Japan; 2 Department of Aeronautics and Astronautics, Graduate School of Engineering, The University of Tokyo, Bunkyo-ku, Tokyo, Japan; 3 Research Center for Advanced Science and Technology, The University of Tokyo, Meguro-ku, Tokyo, Japan; Beihang University, CHINA

## Abstract

In this work, we investigate properties of bidirectional pedestrian streams by studying different experimental datasets from multiple authors. Through the comparison of a scenario where lanes naturally form with two others where lane formation is either obstructed or facilitated, we show the relationship of different pedestrian quantities in regard to the flow ratio (or directional split). On this scope, two measures to account for the degree of congestion and self-organization are introduced. The analysis of the results reveals that the balanced case (where flow is almost equal in both directions) has very peculiar properties which depends on the existence or not of organized lanes and their stability. While the balanced case generally shows the highest level of congestion, this property can quickly change after lanes are formed and when they remain stable. An in-depth investigation revealed that capacity in bidirectional streams is characterized by a dual nature: conflicts with the counter flow and self-organization in lanes. Both aspects have been described using a mathematical model which allowed to define a function for capacity in relation with flow ratio and environmental/cognitive aspects. The expression for capacity proposed in our work agrees with several studies from the literature, eventually allowing to understand the differences among them. We believe our function for capacity enables a more universal treatment of bidirectional streams compared to previous definitions, since it allows to account for steady and non-steady state conditions which represent important mechanisms in their dynamics. The framework introduced here may also help measuring the influence of environmental/cognitive changes in relation with the capacity of bidirectional pedestrian streams.

## Introduction

During the last few decades a growing interest has been shown on topics related with people’s collective motion and pedestrian dynamics was born to describe and predict pedestrian traffic in public facilities. Although research on crowd behavior mostly started as a sociological/anthropological subject [1], in the course of the years a growing interest has been put on quantitative aspects, in particular concerning the assessment of pedestrian traffic capacity. In this regard, the work by Fruin [2] can be considered as a first attempt to provide norms to be used in the design of pedestrian structures. In his work, Fruin introduces the so-called Level of Service (LOS) which is used to grade the comfort of urban facilities from the perspective of pedestrian users.

With the technological improvements of the last decades specifications for pedestrian facilities have drastically improved both in terms of accuracy and range of application. The LOS is continuously updated and additional definitions are provided for new facilities. An increasingly larger part of the The High Capacity Manual [3] is dedicated to pedestrian traffic and its interaction with vehicles in infrastructures such as crosswalks [4, 5]. At the same time, technological improvements that allowed to gather an increasingly larger amount of information on pedestrian motion are making the necessity of tabulated specifications less urgent.

In fact, computer simulation is increasingly used in the design of pedestrian facilities. The most evident advantage of numerical simulation against classical definitions based on specific norms and conventions is the possibility to take into account complex geometries and heterogeneous crowd composition. Although accuracy and reliability of simulations for large scenarios have been debated, computer simulation is surely an efficient tool helping designers to identify dangerous locations and design flaws at the early stages during the development of new facilities [6, 7].

With this said, understanding and defining limits for pedestrian flows still remains an important aspect for the development of safe and comfortable facilities. Accurate simulation models can only be developed if motion of crowds is sufficiently understood and experimental data are fundamental for the validation of those models [8, 9]. In this regard, the progresses in computer simulation have partially downplayed the importance of experimental studies and data-driven approaches are making the need for physical model less stringent. A consequence of this is that the LOS still represents the best approach for grading and categorizing pedestrian spaces. While simple and universal, the LOS has also some limitations, namely the fact that is based on qualitative remarks and the relative small number of infrastructures which are considered in it. For the specific case of the bidirectional flow considered here, only simple and general values are provided for its capacity, although the literature shows that this is a much more complex mechanism.

In addition, since the introduction of the LOS, detection of pedestrian motion has also seen a fast evolution. Nowadays, technologies such as computer vision [10] and distance sensors [11, 12] are commonly used in real situations. Although some studies [13, 14] suggest that such technologies have not reached maturity yet and detection efficiency still depends on crowd density and exposure conditions, there are reasons to believe that in the future more efficient algorithms may contribute in improving their accuracy (in particular regarding tracking capabilities). Also, there are alternatives (e.g. the optical flow) which allow to obtain quantities such as the overall crowd velocities with sufficient accuracy [15].

In this study, we will focus on the bidirectional flow and show that it is possible to study and classify pedestrian motion using criteria related to congestion and self-organization. After studying in detail aspects related with lane formation, we will define a function for capacity using a simple model. The choice of the bidirectional case is related to multiple reasons: it is a very common scenario (corridors, crosswalks, sidewalks or walkways are some examples), it is simple but yet shows emergent phenomena in the creation of organized lanes, it has been extensively studied in the past and it represents the simplest case of bidimensional motion. In this sense, the particular case of the bidirectional flow lies between the widely studied unidirectional motion (e.g. people walking in a circle) and the still “mysterious” motion of crowds in multiple directions (intersections, platform connections, plazas…). To verify the hypothesis introduced in this work and fit the mathematical functions proposed, a large database consisting of pedestrian trajectories from different authors will be used.

This paper is organized as follows: at first, we provide the definitions which will be used in the manuscript; following an overview on previous research on bidirectional flow is given. Later, we will introduce the experimental database and continue by analyzing it using methods presented in the results’ section. Final remarks and considerations for future studies are given in the conclusions.

## Definitions and nomenclature

### Bidirectional flow

Literature is not univocal on the terms used for the bidirectional flow and it is therefore convenient to introduce at first the concepts that will be discussed in this manuscript. We tried to stick to terms generally used in the literature, but in some cases we had to choose between different usages.

To start with, the concept of flow needs to be defined. Taking a corridor as the simplest example, total pedestrian flow is defined as the number of people passing through a given section in a given time. Usually it is measured in (pedestrians) (m⋅s)^−1^ (some old definitions prefer minutes). For more complex geometries (where it is difficult to define a “cross-section”), flow is typically obtained by multiplying density and velocity (usually the absolute value).

The bidirectional flow is characterized by two (monodirectional) streams moving in opposite directions. In case the flow in one direction is bigger than the one in the opposite direction we will refer to the first one as the major flow. The smallest flow in both directions will be defined as the minor flow. When major and minor flows are equal or of similar magnitude we speak of balanced flow. In general, the concept of balanced flow is mostly phenomenological and there is no definition on how similar the streams in both directions need to be in order to call it “balanced”. From a theoretical point of view both flows must be equal to call that configuration “balanced”, but in practical terms any configuration which has fairly similar levels of flow in both directions may be labeled as balanced. In the extreme case of a non-existent minor flow, we will have a unidirectional flow (all pedestrians moving in the same direction). More in general, the asymmetry of bidirectional flows can be measured using the flow ratio (sometimes called directional split) defined as:

$$\begin{array}{c} r=\frac{\text{considered}\;\text{monodirectional}\;\text{flow}}{\text{minor}\;\text{flow}+\text{major}\;\text{flow}}=\frac{\text{considered}\;\text{monodirectional}\;\text{flow}}{\text{total}\;\text{flow}} \end{array}$$ (1)

Note that under the given definition the flow ratio *r* is defined in the interval [0, 1]. In the case of a balanced bidirectional flow it will be equal (or close in practical terms) to $\frac{1}{2}=0.5$. When the minor flow is considered (excluding the unidirectional case) flow ratio will take values in ]0, 0.5[ and in the case of the major flow it will result in values included in ]0.5, 1[. We will refer to the counter flow as the flow in the opposite direction to the one being considered (usually we speak of counter flow referring to the the minor flow).

### Flow regime, phase transition and capacity

To complete the discussion on the fundamental principles of pedestrian dynamics a short remark has to be made on the concepts of flow regime, phase transition and capacity. In vehicular traffic and physics the notion of “state” or “phase” is typically used to describe forms of motion or aggregation. In this work, we preferred to use the concept of “flow regime” given the similarities between fluids and pedestrians’ motion. Although pedestrians represent a particular case and physiological and cognitive characteristics make them different from fluids and cars, we believe that “flow regime” best suits to describe their motion.

In introducing the different concepts, we will base the discussion on the previous literature, which mostly dealt with unidirectional (in many cases strictly unidimensional) motion and used the fundamental diagram (FD) as the main analytical tool. The reasons for using the FD in this introduction are that it is a well-known method in transportation theory and it allows to conceptually define several flow properties with a common framework shared with other disciplines (such as vehicular traffic).

**Fig 1:**
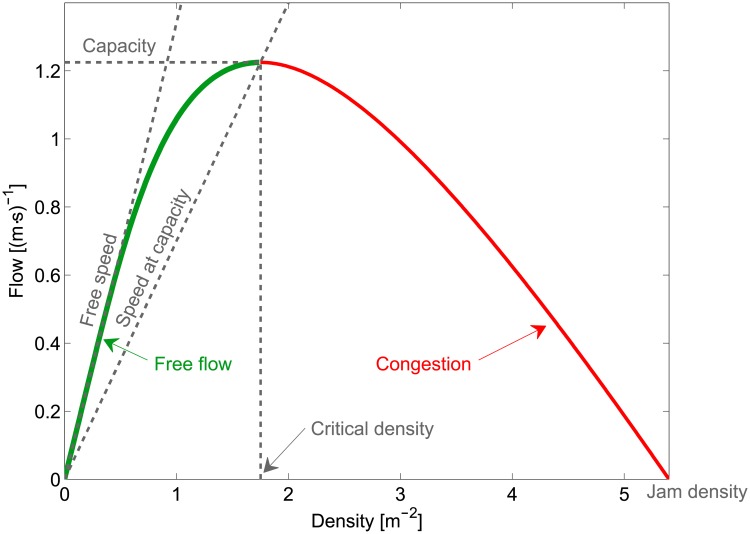
Fundamental diagram. Flow–density representation of the fundamental diagram for pedestrian motion [16]. Numerical values refer to the meta-study by Weidmann [17] and the function proposed by the same author.

From the FD (as the one shown in Fig 1) it is possible to define two flow regimes: free flow and congestion.

Phase transition from free flow to congestion occurs at the critical density, where maximum flow is observed. This particular amount of flow is also defined as capacity and has been the subject of several studies as we will see later. The slope of a line connecting the origin to any single point in the FD provides the velocity at the given flow (or traffic conditions). It is therefore possible to define the speed at capacity, but this value may not be practical in identifying changes in flow regimes since a transition is not seen qualitatively.

In addition, a jam density can be also defined to identify the density where the flow drops to zero and pedestrians are at still (although the validity of this definition is still debated [18] and typically holds true only for theoretical treatments). Jam density can be seen as a limit of pedestrian crowds and therefore we can refer to phase transition only in relation to the passage from free flow to congestion.

It should be remarked that in the case of bidirectional streams there is an additional flow regime which has been often studied in the literature but cannot be recognized in the FD and refers to the organized motion. It is in fact known that under given conditions pedestrians moving in the same direction get organized in groups to reduce the collisions with the counter flow.

The FD has been used in this introduction because it is a well-known concept in transportation and traffic engineering. However, it is important to remark that real measurements of pedestrian properties result in a number of dots dispersed over a large surface roughly defining the behavior indicated as a line in Fig 1. Therefore, clearly determining if a given flow configuration has to be considered congested may become difficult by using the FD alone. For example, Zhang et al. [19] found that fundamental diagrams for unidirectional and bidirectional flow are different. In addition, while the definition of capacity and critical density appears as a single and unique point in the representation of Fig 1, in reality, transition to congestion occurs at a range of densities. In brief, capacity is a rather simple definition from a theoretical perspective, but defining it in practical terms becomes much more difficult. As we will see in the next section, for the bidirectional case considered here the flow ratio plays a central role when investigating the transition to congestion.

Finally, it should be also noted that, in the case of simulation, the deadlock concept is very often used. Some authors used simulations to study the density at which the “jamming transition” occurs, i.e. the density at which deadlocks occur and pedestrians cannot cross the corridor at all. Although this type of studies are important to develop better simulation models, the relation with reality is uncertain since deadlocks almost never occur in real situations and a minimal flow was reported also under very extreme conditions [18, 20, 21] (the origin and nature of this minimum flow is not clear). Therefore, although we will often refer to deadlock occurrence as a phase transition while reviewing simulation models, it is important to remind that this concept do not necessarily applies to real situations (we also noticed that a partially different notation is used for simulation and experimental studies; as a consequence, unifying both terms was not always possible).

## Literature survey

In this section, we will focus on the principal topics of this paper (more specifically bidirectional flow capacity and transition to congestion) and see how past studies have investigated the phenomena. Most of the review will focus on experimental studies, but simulation models have been also used to understand particular mechanisms occurring in bidirectional streams and therefore we will consider them to make the discussion more complete.

### Bidirectional flow capacity

Different researchers already tried to estimate the effect that the counter flow has on the overall capacity of bidirectional flows. Readers interested in a detailed discussion are referred to S1 Appendix which contains an exhaustive review of all works treated here. In this section we will compare only relevant aspects of each study.

**Fig 2:**
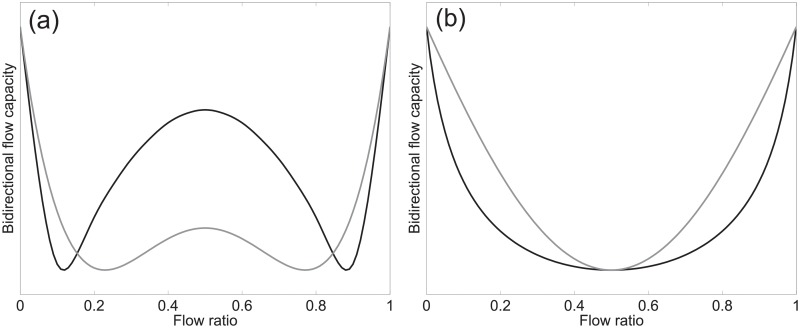
Typical shapes of the bidirectional capacity function. Curves presented here are only intended to support the qualitative discussion of this section, quantitative examples are provided in S1 Appendix. Typical W-shaped capacity functions are given in (a) and U-shaped curves are represented in (b).

One of the main topic of discussion in the past literature regards the relationship between bidirectional flow capacity and flow ratio. Some authors [22–24] claimed that a “W”-shape (like the one schematically represented in Fig 2(a)) describes this relationship, while others [25–29] obtained a “U”-shape (see Fig 2(b) for a schematic example) in their experiments/observations. Some different shapes have been also reported, like the “M”-shaped capacity obtained by Kretz et al. [30].

Table 1 provides a summary of the studies reviewed with their different conclusions on the shape of the capacity–flow ratio relationship. As it can be seen, the considered studies also differ in the numerical values, with some reporting maximum capacities of over 3 (m⋅s)^−1^ and others as small as 1.23 (m⋅s)^−1^. More agreement is found on the minimum value, with most studies reporting values roughly around 1.5 (m⋅s)^−1^, but disagreement is found again on whether the minimum is expected on balanced or unbalanced/unidirectional configurations.

**Table 1:** Capacity–flow ratio relationship: Its shape and minimum/maximum capacity for different studies. Values given in bracket for capacity is the flow ratio at which minimum/maximum is observed; since the relation is symmetric two extrema are always found but only one is reported for convenience.

		Capacity [(m⋅s)^−1^]	
Author(s)	Capacity–flow ratio function	Maximum	Minimum
Navin and Wheeler	W-shaped	1.23 (0.00)	1.05 (0.10)
Cheung	W-shaped	1.53 (0.00)	1.12 (0.01)
Lam et al.	Slightly W-shaped	1.25 (0.00)	1.11 (0.22)
Kretz et al.	M-shaped / Center U-shaped	3.07 (0.10)	1.28 (0.00)
Feliciani and Nishinari	U-shaped	2.20 (0.00)	1.50 (0.50)
Wong et al.	U-shaped	1.62 (0.00)	1.52 (0.50)
Alhajyaseen et al.	U-shaped	2.13 (0.05)	1.34 (0.50)
Zhang et al.	U-shaped	2.20 (0.00)	1.88 (0.50)

In Kretz et al., if bidirectional flow only is considered then minimum is found at the balanced case around 0.5. In Alhajyaseen et al., function tends to infinity for unidirectional flow and it has been therefore computed at a flow ratio of 0.05.

All authors provided phenomenological descriptions to explain why the given shapes were obtained in their studies. Studies presenting a W-shaped capacity function concluded that the high capacity obtained in balanced flows is given by the fact that a clear division is seen among lanes moving in opposite directions and many argued that, under these conditions, the balanced flow could be regarded as two separate unidirectional streams (like if a wall was set among groups of people moving in opposite directions). Many studies in this category concluded that under unbalanced configurations the creation of lanes is more difficult and therefore a small capacity is obtained.

Studies where a U-shaped capacity function was obtained concluded that in the balanced configuration there is a large number of interactions as neither group is dominant on the opposite direction. This results in many people having to adjust their walking direction and it will therefore rapidly lead to a congested motion which reduces speed and finally results in the smaller capacity. The same researchers noted that under unbalanced configurations one direction always dominates the other and therefore interactions are reduced and capacity is higher compared to the balanced case.

To get further clues on why apparently similar studies (all are about bidirectional streams) arrived to different conclusions it is important to compare the context under which observations/experiments were performed. Table 2 contains a more qualitative comparison of the different works by considering the type of study (supervised experiment or on-field observation), the facility studied, its width and whether the flow from both directions was continuous or intermittent (i.e. small groups of people entered an empty corridor and left it empty after their passage, like in the case of a crosswalk with a traffic light).

**Table 2:** Comparison between the different studies. Continuous pedestrian stream indicates a situation in which people continuously flow through the facility, while small group interaction is the case in which two groups limited in size pass each other’s (as in the case of signalized crosswalks).

Author(s)	Type of study	Facility	Width	Pedestrian stream
Navin and Wheeler	Observation	Sidewalk	N/A	Continuous
Cheung	Observation	Walkway	2.5–3.3 m	Continuous
Lam et al.	Observation	Crosswalk	7.2 m / 9.0 m	Continuous?
Kretz et al.	Experiment	Corridor	1.98 m	Small group interaction
Feliciani and Nishinari	Observation	Corridor	6.0–7.4 m	Large group interaction?
Wong et a	Experiment	Corridor	3.0 m	Small group interaction
Alhajyaseen et al.	Observation	Crosswalk	4.0–10.0 m	Small group interaction
Zhang et al.	Experiment	Crosswalk	4.0 m	Small group interaction

In Lam et al. the term “crosswalk” suggests an interaction between small groups, but the images provided by Lam et al. [24] implies that possibly the flow was rather continuous. In Feliciani and Nishinari pedestrian stream was mostly intermittent but interacting groups of pedestrians were rather large thus making the flow continuous at certain times.

By comparing Tables 1 and 2 it is interesting to see that there seems to be a correlation between the type of pedestrian stream and the shape obtained for capacity. In the case of continuous streams a W-shape was typically obtained, for small group interactions a U-shape seemed more common (with the partial exception of Kretz et al. who concluded that unidirectional flow is always less efficient). Facility’s width apparently has little or no relation with the shape of the capacity function as well with the reported maximum and minimum values. We could argue that facilities having a width in excess of 3 m are comparable in terms of the phenomena observed.

A tentative explanation for the different shapes of the capacity functions, also considering the different contexts, may be the following. When there is no clear distinction between the minor and major flow, lane formation is less efficient, since it is more difficult to determine which group is dominant and should be allowed to take a given section of the corridor/facility. This behavior could be particularly strong for small groups of pedestrians, who have only a short time to interact and determine which group should take which part of the corridor. Under these conditions the “chasing” behavior (seen in people moving in the same direction) is less effective since it is not clear who should be followed to reduce collisions. However, over the long run, especially when pedestrians continue to flow at stable conditions, lanes could form separating both directions. In facilities where pedestrians are constantly present, lanes may never disappear throughout the day and may follow traffic regulations. Under these conditions pedestrians entering the facility already know which side/part of the corridor is “dedicated” to their direction. This could be the mechanism leading to the transition into a W-shape function as reported in long time observations. In the study by Kretz et al. a transition from a “V” to a “U” shape is seen during the experiment (see S1 Appendix for details) and it can be further speculated that a “W” would be obtained if the experiments were run for a longer time.

### Phase transition

As we noted, in the bidirectional case, transition to congestion is different from the simple unidirectional case; the phenomena occurring are more complex and therefore it is difficult to consider the fundamental diagram alone. Here, we will see how past studies have investigated phase transition in bidirectional flows. Most of the studies used numerical simulation, which allows to easily vary conditions and study scenarios involving a large number of people in comparatively short time.

One of the most complete study on the subject of phase transition has been presented by Nowak and Schadschneider [31], who have used a common Cellular Automata (CA) model to assess the stability of lanes and study more in detail the transition between different flow regimes. In their study, four different flow regimes are considered: free flow, disorder, (stable) lanes and gridlock. Nowak and Schadschneider mostly considered periodic boundary conditions (i.e. pedestrians leaving from one side of the corridor are reintroduced on the opposite side, corresponding to a loop in reality), but Weng et al. [32] also extended the analysis to the case of open boundary conditions (pedestrians leave from the respective side and vanish from the system). For the latter case, they concluded that for certain densities, only two conditions are observed: free flow and perfectly stopped phase (or deadlock). The model by Weng et al. had the particularity to consider pedestrians with different walking speeds and allowed them to observe that “in the stage of lane formation, the phenomenon that pedestrians exceed those with lower walk velocity through a narrow walkway can be found”, which later led them to the conclusion that transition is driven by spontaneous fluctuations which turn the first metastable state into congestion.

Muramatsu et al. used a lattice gas model with periodic [33] and open [34] boundary conditions to determine the density at which the low density free flow changes into a high density deadlock. However, in contrast to Nowak and Schadschneider and Weng et al., in their model the transition did not include an organized state with stable lanes and therefore it is not representative of real conditions. Tajima et al. [35] employed a similar model with open boundary conditions to also study the jamming transition (or deadlock formation). In the case of Tajima et al. there is an abrupt transition from motion in lanes to deadlock and free flow is not considered.

Alonso et al. [36] created a continuous model which calculates pedestrian motion from Newton’s second law, taking into account viscoelastic contact forces, contact friction and ground-reaction forces. Their model focus on extreme phenomena and takes therefore into account three different regimes which are identified as lane formation, avalanches and clogging. Based on the results of their simulations, Alonso et al. concluded that phase transition should occur earlier, or at lower densities, for the balanced case.

## Research questions and objectives of this study

In light of the discussion on the past literature and the current state of research on bidirectional pedestrian streams, this study aims at investigating several points as listed below:

- Whether the capacity–flow ratio function should be described using a “U” or “W” shape and why apparently similar studies arrived to different conclusions.
- Whether the capacity function for bidirectional flow can be considered universal or it depends on different factors. If so, which factors are relevant in determining its shape.
- Which equation can be used to describe its shape and if there is any model which can help obtaining it (without simply using a common function to fit empirical data).
- How is it possible to predict if a bidirectional stream will turn into congestion (without using the fundamental diagram) and which quantities are relevant in this regard.

## Experimental data and methods

### Experimental data

To investigate more in detail properties of the bidirectional flow and try to answer the above research questions, we created a database consisting of different experiments performed by several researchers [19, 37–41]. This section will be devoted in explaining those experiments and the methodology used to categorize them into different groups. We will try to limit the details and go straight to the points relevant for this work; readers interested to more specific aspects of each study are referred to S2 Appendix.

Although there is already a large number of data available on pedestrians trajectories, the type of analysis considered in this work requires very accurate data and therefore the number of usable dataset shrinks quite quickly. In general, criteria employed for selecting a specific dataset for inclusion in the final database were based on scientific suitability (in respects to the objectives of this study), quality requirements and reliability of the source. As a consequence, only datasets considering cases of well-delimited bidirectional flow have been selected and we tried to cover a range as large as possible in regard to density and flow to increase the universality of the conclusions.

At the end, only supervised experimental studies could be used, all of them recorded using cameras and later processed using the PeTrack [42, 43] software to extract trajectories. Depending on camera and markers configurations an accuracy of up to 1 cm can be achieved by extracting trajectories with PeTrack. While technical details (markers used, trajectory extraction method, camera resolution and frame rate) and geometrical aspects (corridor width and length) are very similar among the experiments considered, there are large differences on the experimental procedures. We consequently decided to divide the datasets into 3 categories, which will be also relevant in relation to the objectives of this study.

**Fig 3:**
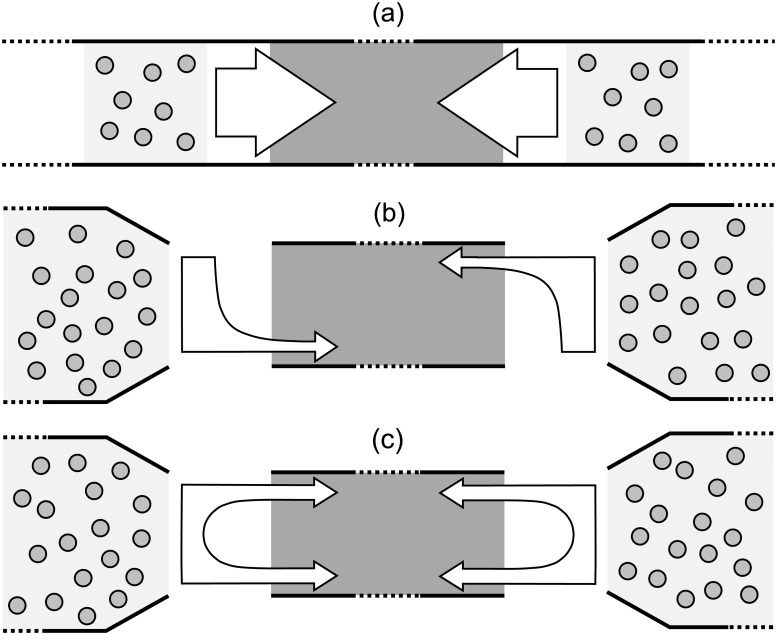
Types of bidirectional flows considered in this work. (a) represents a class of supervised experiments with a limited number of participants naturally interacting while walking through a long corridor (people kept walking even after passing the central area). No specific instructions were given on how to behave but participants were asked to simply walk. (b) represents experiments with a larger number of people flowing into a shorter corridor for 1–2 minutes with free choice to leave either side (right/left) at the exit. In this case, participants were allowed to exit the corridor from the side they liked and experiments were repeated under very similar conditions. Most people left from the right side as experiments were repeated (probably because of their daily custom). (c) represents a class of experiments where half of the participants were asked to exit from the left side and half from the right side. Strong interactions were observed as lane formation was clearly obstructed due to the specific instructions given.

**Fig 4:**
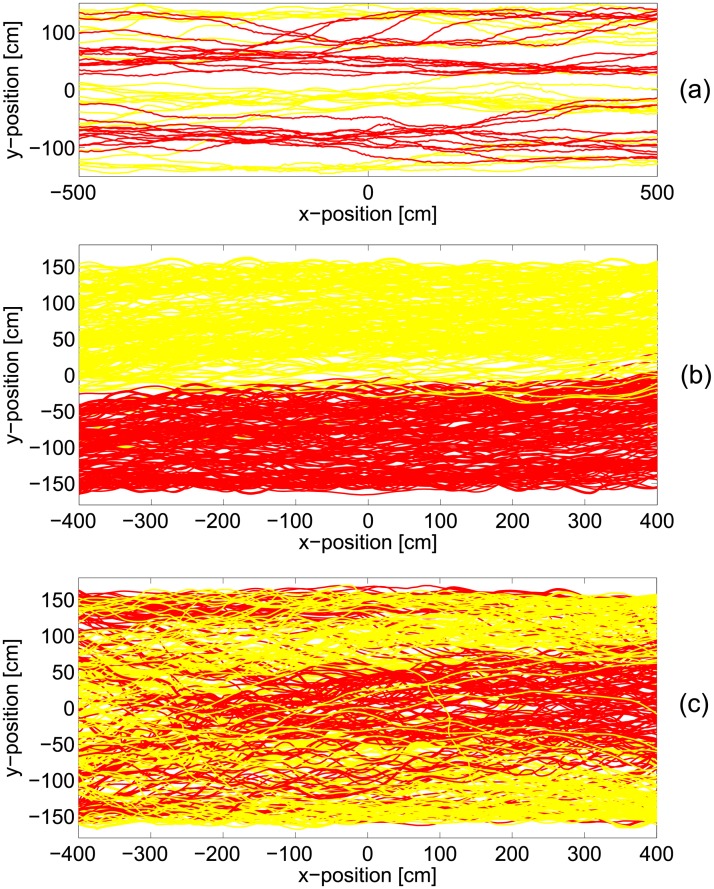
Bidirectional flow trajectories. Example of trajectories from some of the cases considered in the three groups. For each case several repetitions were performed and the example provided here is relative to only one execution; all are relative to the balanced configuration. Also, total time considered for plotting trajectories is different, so the number of lines is not relative to the density of pedestrians. Yellow trajectories are relative to left to right walkers, red to the opposite direction.

Table 3 summarizes the most remarkable differences among the 3 types of study considered, Fig 3 provides a schematic description of the experimental procedures/observed phenomena and Fig 4 shows some typical trajectories relative to each group of experiments.

**Table 3:** Grouping of experimental datasets depending on the type of bidirectional stream considered and the instructions given to participants.

		Single run		
Group	Case study	Time	People	Instructions
(a)	Natural small group interaction	10–20 s	≈ 50	No instructions
(b)	Facilitated lane formation (learning process)	1–2 min	≈ 300	Free to choose leaving side (left/right)
(c)	Strong obstruction with forced motion	1–2 min	≈ 300	Leaving side determined (no choice)

Datasets considered in group (a) consist of experiments with a relatively small number of pedestrians walking in a more or less natural environment and without any specific indication on destination or behavior to be followed. A mock corridor was set up over a long distance and participants kept walking in their respective directions after passing each others’ (people had to walk “straight” for about 20-30 m although only the central 10 m are used for data analysis). In those experiments, groups were shuffled during each execution and therefore participants were not able to get any advantage from the multiple repetitions. It is therefore excluded (or very unlikely) that participants were able to develop some form of organization evolving over the time of the experiment (i.e. a long term cognitive process).

In (b), participants also had some behavioral freedom; in particular they could freely choice from which side of the corridor to leave after passing through it (in this case people where able to “exit” the corridor after walking in it for about 10 m). This experiment was repeated under very similar conditions: after the first execution, two lanes clearly formed dividing both flows and participants occupied the right side in each direction (experiments of these datasets were performed in Germany which is a right-driving country). At the second execution, participants already learned that forming two lanes would make crossing of the corridor easier and they continued showing this behavior until the last repetition. In some sense the case considered in (b) can be seen as a loop, since each repetition is virtually related with the previous runs.

In the experiments of group (c) a very different experimental procedure was used: each pedestrian was asked to leave the corridor from a different side (half left and half right). Therefore, although experiments were repeated under similar conditions, pedestrians did not get any advantage by knowing the experimental setup. In this case lanes could not form (or did not last for a long time) and a sort of diagonal motion was observed, with people entering from the middle of the corridor and trying to exit from both sides. This was the case with the strongest interactions and people obstructing each other’s were observed very frequently.

### Analytical approach and computational methods

**Fig 5:**
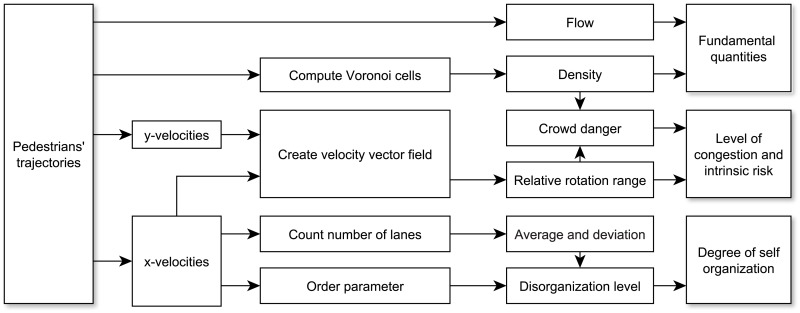
Computational method and relevant quantities. Summary of the quantities considered in this study and methods used to compute them.

This section will present the computational methods used in the analysis of the experimental database presented above. In discussing the details, Fig 5 can be used as a reference to understand the relevance of each quantity in regard to the overall study.

In general, the analytical approach is based on our previous research (see in particular [37, 44]) and on studies from other researchers (for example the works by Saberi et al. [45, 46] have been a source of inspiration). The overall process for analyzing pedestrians’ trajectories can be summarized as follows: each case will be divided into small time intervals and for each interval several quantities relative to the smoothness of motion and the degree of self-organization will be computed. Simple operations like velocity or flow calculation will not be described here as they belong to basic methods established in the literature. Density calculation using Voronoi cells has also been used for several years and it is a well-known procedure in pedestrian dynamics and readers may refer to [47] for details.

Before considering more detailed aspects, we need to clarify the difference between the concept of congestion and organization, which will be central points in the following discussion. The concept of congestion is closely related with the one of capacity (as already discussed in the introduction). When a crowd can move for an indefinite time under constant conditions, then the motion can be defined as uncongested. If, for some reasons, it is not possible to keep moving at a constant pace, then we can say that congestion occurred. The concept of organization is slightly different. In a bidirectional flow, a crowd is defined as organized when lanes are clearly defined and interactions occurs only with people moving in the same direction. For instance, a unidirectional flow is by definition organized, but may be congested when the density is too high to allow a smooth motion. Also, a well organized bidirectional stream may become congested if people start overtaking within their lanes. Of course a well organized crowd could help reducing congestion, but this may not be a sufficient condition.

#### Degree of congestion and intrinsic risk

To determine the degree of congestion (or in other words the smoothness of motion), the so-called “relative rotation range” has been used and its calculation will be described as follows. The computational method is analogous to the recently proposed “congestion level” [44], with the only exception that in this work we are using the whole experimental section as the Region of Interest (ROI).

**Fig 6:**
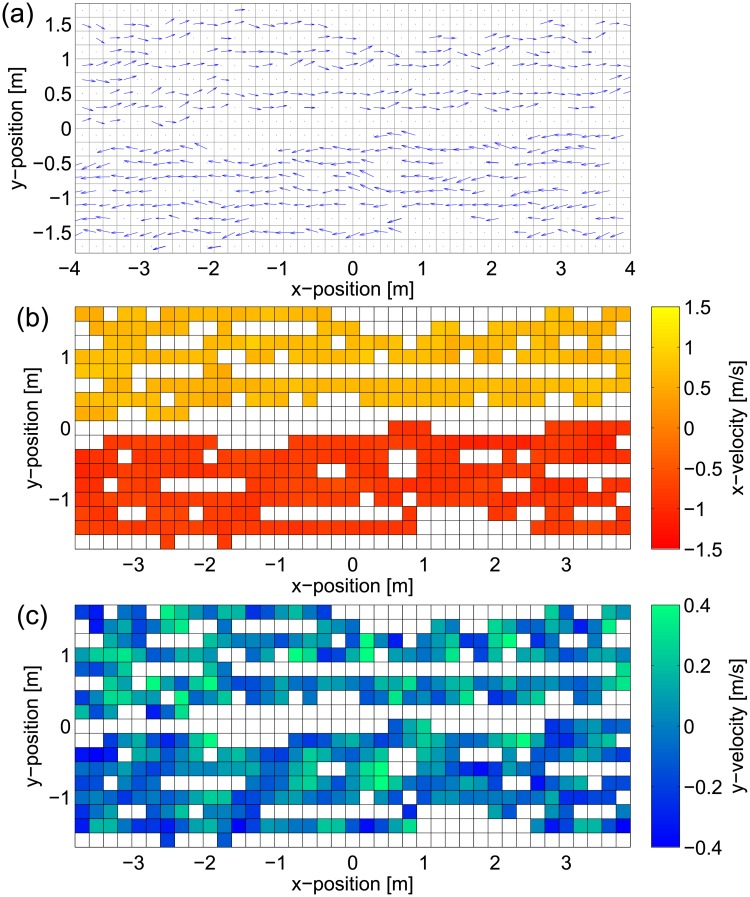
Velocity representation of bidirectional streams. Different ways of representing pedestrian speed in the bidirectional flow; the example provided here corresponds to one experiment of dataset D, figures are generated for a 2.5 s time interval and a 0.2 m mesh size. The 3 representations are: (a) vector field, (b) *x*-velocity (direction of motion) and (c) *y*-velocity (lateral motion).

To start with, the surface of the corridor (or the section to be studied) is divided into a mesh to allow the computation of the average velocity in *x*- and *y*-direction relative to the overall crowd motion in a given time interval, thus creating a velocity vector field like the one presented in Fig 6(a). A mesh having 0.2 m in side length and a sampling time interval of 2.5 s were chosen to allow an accurate description of pedestrian motion under different densities [44].

The rotation range is a measure for the amount of rotation observed in a pedestrian stream derived from the concept of vorticity commonly used in fluid-dynamics. This concept is inspired from our previous research, in which we found that rotation is an important mechanism both on the individual [38] and crowd level [37, 48] and the work by Helbing et el. [18] who identified “crowd turbulence” as a cause for accidents. As described above, the average velocity is computed in each cell and for each time interval, thus resulting in a vector field $\vec{V}\left(x,y\right)$ containing velocities for the cells where pedestrians have transited. Taking the rotational of this vector field will result into:

$$\begin{array}{c} \vec{R}\left(x,y\right)=(\begin{array}{c} r_{x}\\ r_{y}\\ r_{z} \end{array})=\vec{\nabla }\times \vec{V}\left(x,y\right) \end{array}$$ (2)

where *r*_*x*_ and *r*_*y*_ are 0 since *x*- and *y*-velocities lies on the same plane. As a consequence, information on the amount of rotation is contained in *r*_*z*_ only. To make no distinction between clockwise and anti-clockwise rotation the difference max(*r*_*z*_)−min(*r*_*z*_) is taken over the whole vector field and this difference is defined as the rotation range. When the flow is uniform small values are obtained, with the rotation range growing with the degree of congestion. In other words, the rotation range can be used to judge if pedestrians are moving in a congested way or not. To also account for global velocity fluctuations the average velocity is included, thus defining the relative rotation range as:

$$\frac{\text{max}(r_{z})-\text{min}(r_{z})}{\left|\vec{v}\right|}$$ (3)

The relative rotation range allows to define the level of congestion in the experimental area considered and consequently set a threshold for capacity. This aspect will be discussed in detail later while presenting the results. Based on the relative rotation range it is possible to define a further quantity named “crowd danger” which defines the intrinsic risk in pedestrian crowds [44]. This is simply obtained by multiplying the relative rotation range with the density, thus assuming that the risk related with crowd motion increases with both density and degree of congestion.

#### Degree of self organization

To define the degree of organization, there are several quantities which can be used in the case of the bidirectional flow. For example, Duives et al. [49] provides an interesting comparison between three different measures using trajectories resulting from a laboratory experiment and several simulations for the case of bidirectional flows. In this work, we will consider both the average and the variation in the number of lanes and a quantity derived from the combination of them with the order parameter.

**Fig 7:**
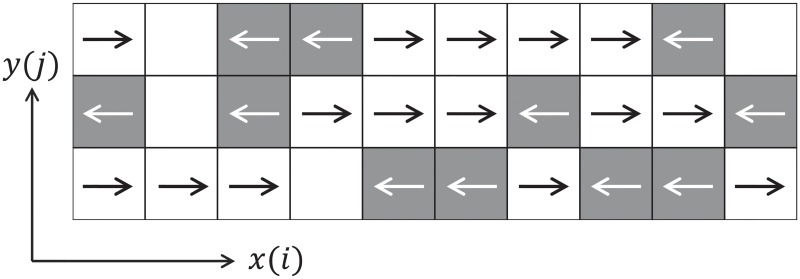
Order parameter. Schematic representation used for the calculation of the order parameter. Each cell indicates a positive or a negative *x*-velocity.

The average number of lanes is simply obtained by counting them in each *x*-position of the previously introduced grid. A lane is defined as a set of cells having the *x*-velocity in the same direction. Cells containing no information are ignored. The case provided in Fig 6 shows a typical example of a 2-lanes structure. In more chaotic situations (like the hypothetical case illustrated in Fig 7) it is possible that the number of lanes is not the same everywhere and therefore defining an overall average and its deviation may become useful in the analysis.

However, both quantities are not sufficient to evaluate the degree of organization since a chessboard-like structure (very unlikely but still possible) will result in a zero deviation, thus mistakenly indicating an organized structure in lanes. To avoid this problem, the order parameter will be also used in the analysis.

The order parameter has been often used to assess the stratification (or degree of organization) of systems composed of lanes, with applications ranging from colloidal fluids [50] or chemical processes [51] to also include the case of pedestrians [31]. The calculation of the order parameter starts by computing *ϕ*_*j*_ for each row *j* (referring to Fig 7):

$$\begin{array}{c} \phi_{j}={\left(\frac{n_{left}-n_{right}}{n_{left}+n_{right}}\right)}^{2}={\left(\frac{n_{left}-n_{right}}{n}\right)}^{2} \end{array}$$ (4)

where *n*_*left*_ and *n*_*right*_ is the number of cells moving to the left and right direction respectively. The sum of *n*_*left*_ and *n*_*right*_ obviously leads to the total number of cells *n*. After computing *ϕ*_*j*_, the global order parameter can be obtained by considering all rows as follows:

$$\begin{array}{c} \Phi =\frac{1}{m}\sum_{j=1}^{m}\phi_{j} \end{array}$$ (5)

where *m* is the total number of rows. The order parameter is by definition never negative and it is generally bigger than zero also in the extreme case of random motion (the order parameter can become zero for perfectly aligned columns, but this configuration is never observed in real situations). For instance, it could be inferred (a validation using a Monte Carlo method is provided in S3 Appendix) that the expected value for the order parameter in a random configuration having flow ratio *r* is:

$$\begin{array}{c} \left\langle \Phi \right\rangle \;=4(1-\frac{1}{n})\cdot r\cdot \left(r-1\right)+1 \end{array}$$ (6)

where *n* is again the number of columns. This clearly shows that there is a dependance between the order parameter and the value we may get for a particular configuration having flow ratio *r*. In other words, the order parameter does measure the degree of stratification, but does not allow to assess the organizational performance in respect to a random configuration. To overcome this limitation we will define a measure for the disorganization level by combining the order parameter with information on lanes as:

$$\begin{array}{c} \frac{\text{Var}(N_{lanes})}{\text{Avg}(N_{lanes})\cdot \Phi } \end{array}$$ (7)

where Φ is simply the measured order parameter and *N*_*lanes*_ the number of lanes in the different *x*-positions. Although defining a measure for disorganization may seem unconventional (a measure for organization may seem more logical), the present definition was preferred as it becomes zero for perfectly organized structures (the inverse measure for organization would tend to infinity).

## Experimental results and discussion

We will now discuss the results and see how the different properties of bidirectional streams change depending on the amount of flow in each direction (and also in respect to the flow ratio). Each section will cover one aspect of the diagram presented in Fig 5 for the three groups considered in Table 3.

### Fundamental quantities

**Fig 8:**
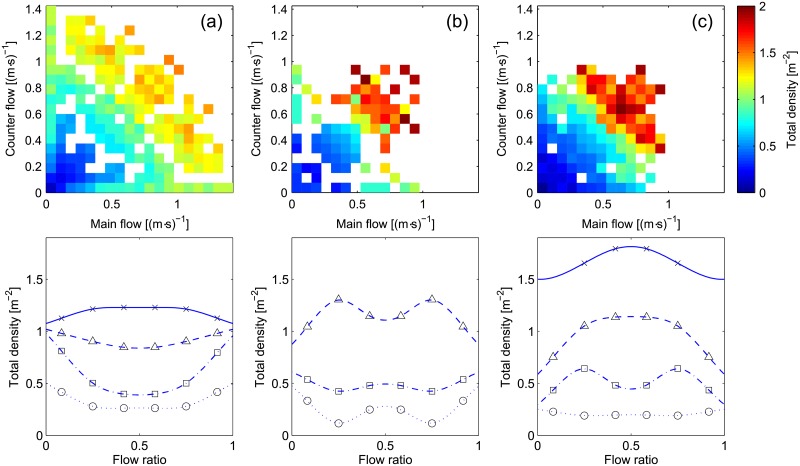
Density. The same color scale is used for all scatter plots. Interpretation for the flow ratio representation is provided in Table 4.

To start with, we can consider the density, which is one of the most important and most widely discussed property of pedestrian crowds. Fig 8 presents different representations for density for each of the scenario considered in this study. The scatter plot allows to examine the relationship between density and directional flow in a bidimensional way. Given the amount of flow in one direction and the relative counter flow, the average density obtained during the multiple experiments is given. A resolution of 0.075 (m⋅s)^−1^ has been chosen to allow providing an overall clear yet precise representation.

To make the interpretation of the results easier and allow to understand the importance of flow ratio, an additional representation is provided. In the lower part of Fig 8 the average density relative to a particular total flow and flow ratio combination is provided in the form of graph. The line representation can be simply obtained from the scatter plot considering that for a given point the total flow is the sum of both axes while flow ratio is the angle formed with the abscissa. From this point of view, it should be clear that to correctly reproduce the results in a line, different curves for the different levels of flow are necessary. A legend for the different symbols used in Fig 8 is provided in Table 4. The same symbols will be used throughout this section when presenting the results for different quantities. Finally, to make visualization easier a common smoothing spline is used to connect the different points.

**Table 4:** Legend for symbol and line styles used in presenting the results in relation to flow ratio.

Flow level	Total flow	Symbol	Line style
Low	0.0–0.4 (m⋅s)^−1^	Circle	Dotted line
Medium	0.4–0.8 (m⋅s)^−1^	Square	Dash-dot line
Considerable	0.8–1.2 (m⋅s)^−1^	Triangle	Dashed line
High	1.2–1.6 (m⋅s)^−1^	Cross	Solid line

The results presented in Fig 8 clearly show that there is an evident difference between the three scenarios considered. Maximum densities are higher in both group (b) (facilitated lane formation, in the center) and group (c) (strong obstruction, on the right), which is obviously related to the highest number of participants and the longer execution of each repetition. In case (b) the solid line relative to high levels of flow is missing since data were not sufficient for an accurate description. From the scatter representation it is possible to notice that, in particular for case (a), there are three regions which define similar levels of density. A low density area for a total flow below around 0.5 (m⋅s)^−1^, a high density area for flow above around 1.2 (m⋅s)^−1^ and an area in the middle.

However, effect of total flow and flow ratio becomes more evident when the line representation is considered. In case (c), where obstruction is created on purpose, it is seen that density is higher for the balanced configuration and it increases as the total flow gets higher. In case (b), which had a procedure making the creation of organized lanes easier, a different picture is portrayed, with the density in the balanced case being similar to the levels of unidirectional motion (except for the case with low levels of flow, where it is not possible to talk of “lanes” since densities are too low). This may show that, as some authors said, an organized bidirectional flow is equivalent to separated unidirectional streams moving in opposite directions. Finally, it is interesting to notice the transition occurring in case (a), which represents a natural interaction where lanes form and dissolve. At low and medium levels of flow the density is clearly lower in the balanced case, but the inverse situation is observed for the high-flow condition. In this case, density seems to level up as total flow increases.

### Degree of congestion and intrinsic risk

**Fig 9:**
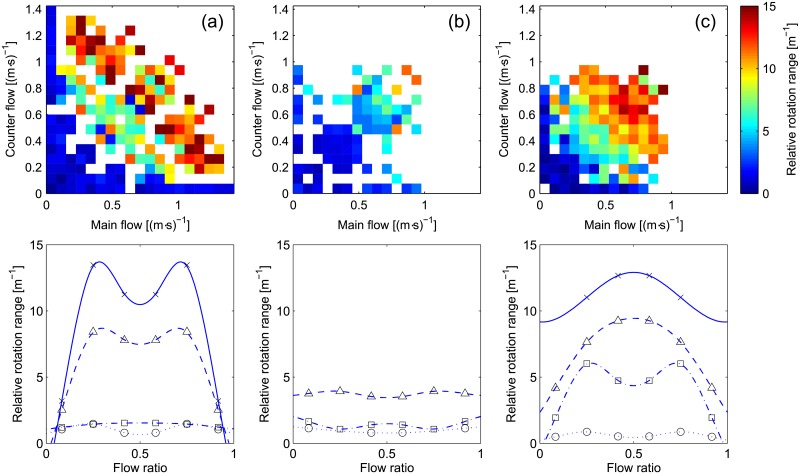
Relative rotation range. The same color scale is used for all scatter plots. Interpretation for the flow ratio representation is provided in Table 4.

We can continue the analysis by considering more complex quantities, with the results for the relative rotation range given in Fig 9.

From Fig 9 it is seen that case (b) has very low levels congestion and the effect of flow ratio appears minimal. This is due to the fact that lanes existed for most of the experiment’s duration and were readily formed at the beginning. Under these conditions flow ratio is mostly a measure describing the difference in size between both lanes and qualitative aspects are homogeneous. Case (c) clearly shows that if lanes are not allowed to form then the balanced case is the most congested regardless on the level of flow (except for very low levels). Case (a) again shows some sort of transition and we could argue that the reduction in congestion observed in the balanced case may be a result from the self-organization in lanes. Nonetheless, relative rotation range for cases (a) and (c) has similar values at high levels of flow, meaning that even if lanes form in case (a) the motion within them tend to be quite unstable.

**Fig 10:**
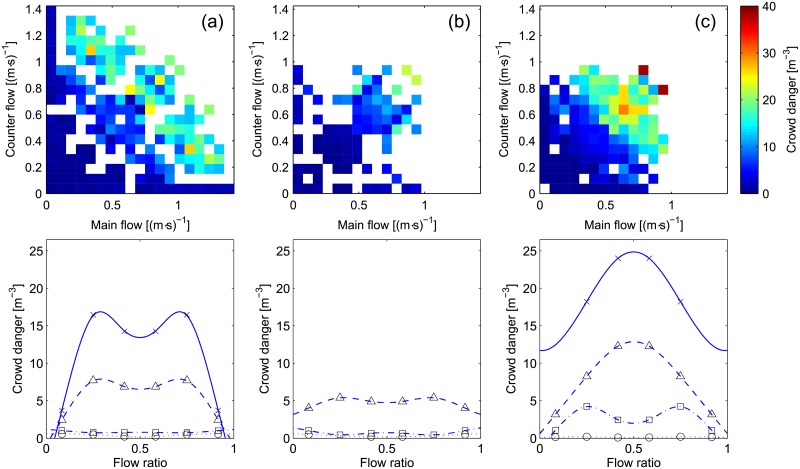
Crowd danger. The same color scale is used for all scatter plots. Interpretation for the flow ratio representation is provided in Table 4.

But to allow a more consistent comparison between the various cases, the crowd danger (which was defined as the product of density and the relative rotation range) can be used, with results provided in Fig 10.

The crowd danger depicted in Fig 10 puts the discrepancies between case (a) and (c) under a different perspective. Although (a) and (c) had similar levels of congestion, the highest densities of case (c) create a higher potential risk for the moving crowd. The combination of density and congestion makes the effect of the flow ratio more evident and the balanced case may be considered two times more dangerous compared to the unidirectional motion. On the other side, the reduction in congestion observed in case (a) in the balanced case appears less important when put into the perspective of potential risk. This may show that lanes do not only need to form but also need to be stable.

### Degree of self organization

**Fig 11:**
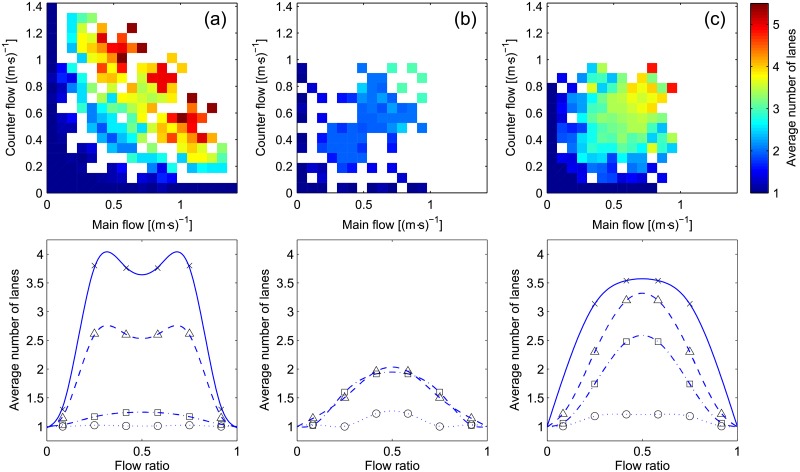
Average number of lanes. The same color scale is used for all scatter plots. Interpretation for the flow ratio representation is provided in Table 4.

To start the discussion on the degree of self-organization, we may consider the average number of lanes, which is a very simple and fundamental characteristic of bidirectional streams. Results for the number of lanes are given in Fig 11 using the usual format.

Case (b) represents probably the simplest case to consider. Since formation of lanes was facilitated by the experimental procedure, a maximum of 2 lanes are formed in the balanced configuration. Unidirectional flow (flow ratio 0 and 1) obviously form only one lane. In unbalanced configurations lanes also formed but density was much lower in one direction, thus creating long strips of unidirectional motion, which ultimately led the number of lanes to take values between 1 and 2. In case (b) it is also important to notice that discrepancies between different levels of flow are minimal, thus suggesting that lanes formed straight away from the very beginning of the experiment.

The picture is clearly different in (c), where the number of lanes grows with the total flow and reaches a maximum which is well above 2. This shows that the more people entered the corridor the more complex became the organization and the balanced case clearly performed the worst in every condition. It is also interesting to see that the curve for the average number of lanes follows a sort of inverted parabola for all levels of flow.

Finally, case (a) shows again a sort of transition: at low levels of flow an inverted parabola is repeated with the balanced case having the highest number of lanes, but as soon as the flow reaches higher levels some sort of self-organization is seen which contributes in reducing the average number of lanes. It has to be remarked, however, that the number of lanes for case (a) is higher than any other case and this despite the relatively smaller width of the corridor. So, while the number of lanes may give some clue on how much organized crowds were, another measure is required to understand better this aspect.

**Fig 12:**
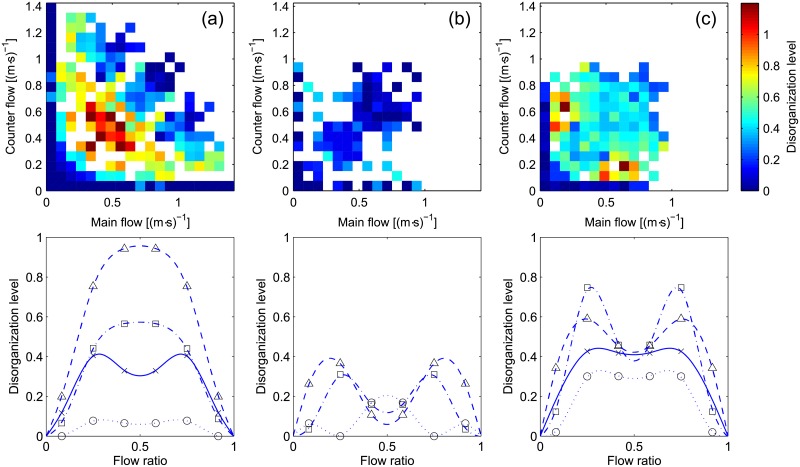
Disorganization level. The same color scale is used for all scatter plots. Interpretation for the flow ratio representation is provided in Table 4.

In this regard, we can now consider the disorganization level, whose results are given in Fig 12.

A first glance at Fig 12 reveals a quite different picture from what observed earlier. In particular, case (a) presents a disorganized region for moderate levels of flow which reaches the maximum around the balanced configuration. Since case (a) represents a situation where two groups of people encounter each other’s in a corridor, the flow during the whole process can be seen as a line originating from the origin and having the slope relative to the flow ratio. The disorganized region represents the moment when lanes form and dissolve, while the organized region seen for high levels of flow is relative to the motion in lanes after groups already formed. From case (a) it is learned that a disorganized and partially congested phase is seen when lanes are being formed, but, if their formation succeed degree of congestion decreases and the level of organization increases.

Case (b) presents a quite homogenous scenario, with low levels of disorganization seen almost everywhere. Unbalanced configurations tend to be less organized, but data also tend to be scarce for those configurations. The results for case (c) show that when lanes do not form (or are not allowed to form), then the level of disorganization remains moderate independently on the total flow. In (c) it is also interesting to notice that the balanced case is disorganized independently on the level of flow. This shows that when lanes do not form, the balanced case does not show an improvement. On the other side, unbalanced configurations tend to be disorganized at low levels of flow but the difference with the balanced case get minimal as the total flow is increased.

To summarize, in the analysis above which involved several quantities, we generally confirmed what different researchers speculated in regard to the bidirectional flow: when lanes form the bidirectional motion is split into several streams moving in unidirectional way. Our results clearly showed that in the balanced configuration lower levels of congestion and an higher degree of self-organization are reached when lanes are formed. If lane creation is made difficult by external effects (which in this case was related to the experimental procedure) than the balanced case is similar to unbalanced configurations. We also found that in the steady-state cases (i.e. where conditions did not change during the experiment) an inverted parabola generally describes the average number of lanes.

## Numerical modeling of capacity

Based on the qualitative and quantitative results from the previous section we will model different aspects of bidirectional flow, finally leading to the definition of a function for capacity taking into account the transient effect of lane formation.

In the above discussion and the literature overview we have seen that bidirectional flow capacity is in general not linear. In our previous research [37], we observed that anticipation is very weak when both groups of pedestrians encounter in counter flow situations and other studies described the motion of pedestrians in packed conditions as a percolation process in which pedestrians “diffuse” through the counter flow [25, 52]. Although the densities relative to the transition into congestion are not extremely high, we can assume that similarities exist when it comes to the formation of congestion and its relation with flow ratio.

**Fig 13:**
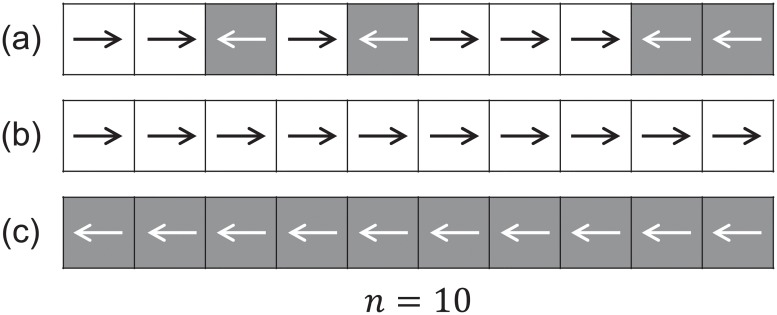
Theoretical grid for estimating the effect of counter flow. Examples of row configurations with *n* = 10 cells, gray cell indicates counter flow. In case (a) it is not possible to pass from one side to the other in neither direction. Case (b) and (c) represent “open” cases in the right and left direction respectively. Please note that letters used here do not refer to the 3 experimental groups considered earlier.

Under these conditions, the corridor (or section) where pedestrians walk can be considered as a grid like the one shown in Fig 13. We will define the main flow as moving from left to right (white cells in Fig 13) and the counter flow moving in the opposite direction (gray cells). In each cell, a pedestrian is assigned to the counter flow with a probability *r* (the probability of belonging to the main flow is therefore 1 − *r*). In more practical terms (and to link it with the previous discussion) we can call *r* the flow ratio.

In a number of models from different disciplines which consider interactions among cells. Examples are the Ising model for ferromagnetism in physics or the several variants of vehicular models used for traffic engineering, many of them considered within the broad context of Cellular Automata. In addition, Yanagisawa [53] considered a game-theoretical approach to model interaction among cells for the case of bidirectional flow. In our model, cells are independent and the behavior is purely stochastic. We will see that although the model presented here is quite simple, it is sufficient to describe qualitatively the more complex mechanisms occurring in bidirectional pedestrian streams.

**Fig 14:**
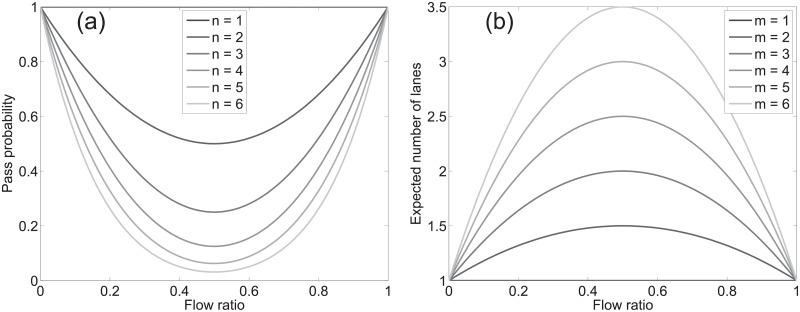
Open path probability and expected number of lanes. Different grid sizes are considered.

We can define the “open path” probability as the probability to have all cells pointing in the same direction (either left of right). Fig 13 shows some examples for possible configurations. It could be inferred (a numerical validation is given in S3 Appendix) that the probability of having an “open path” in either direction is:

$$\begin{array}{c} p_{open\;path}\left(r,n\right)={\left(-1\right)}^{n}\cdot [{\left(r-1\right)}^{n}+{\left(-r\right)}^{n}] \end{array}$$ (8)

with *n* being the number of cells. Fig 14(a) shows the plot of Eq (8) for *n* = [1, 6]. As it can be intuitively guessed, the minimum is found at $\frac{1}{2}$ (i.e. balanced configuration).

Since we want to use this function to model capacity, we need to consider some transformations to adapt *p*_*open path*_ to real data. We can therefore define as *q*_*max*_ the maximum capacity and this will be equal to the capacity for unidirectional flow (2.20 (m ⋅ s)^−1^ will be used here). The lowest transition is happening at the balanced flow configuration, so, as a consequence, the value *q*_*min*_ will be assigned to a flow ratio $\frac{1}{2}$. This leads to the following form for the function describing capacity:

$$q_{tot}\left(r,n\right)=p_{open\;path}\left(r,n\right)\cdot \alpha +\beta$$ (9)

$$\beta =\frac{q_{min}-p_{min}\cdot q_{max}}{1-p_{min}}$$ (10)

$$\alpha =q_{max}-\beta$$ (11)

where *p*_*min*_ is equal to $p_{open\;path}\left(\frac{1}{2},n\right)$ and corresponds to the minimum value of *p*_*open path*_. This expression may be useful to model the overall dynamics toward a chaotic counter flow, but we know that humans do have the capability to organize themselves in lanes.

**Fig 15:**
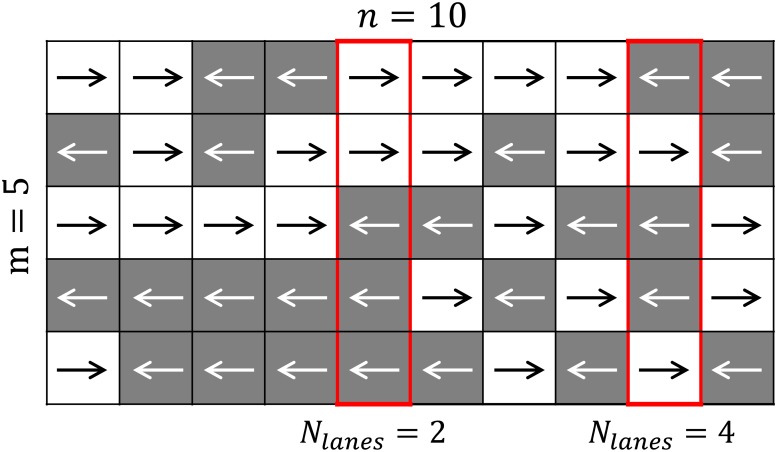
Grid used for the calculation of lanes. Bidimensional grid representing pedestrians moving in opposite directions in a counter flow. Lanes are counted for the two selected boxes.

The formation of lanes plays an important role in bidirectional streams and the average number of lanes had an inverted parabola. To model this aspect we can now consider a bidimensional grid with similar characteristics to the model considered to compute the open path probability. We may now consider a model consisting of *n* × *m* cells as the one shown in Fig 15. The theoretical model discussed here is conceptually similar (and inspired) from the grid considered in the above experimental discussion (see Fig 6(b) and 6(c)), although cell size is irrelevant here and only its number is considered. As for the previous case, each cell is assigned to left or right direction with probability *r* (being the flow ratio).

We now want to know what is the expected number of lanes 〈*N*_*lanes*_〉 given the flow ratio *r*. As we know, a lane is typically defined as a group of people walking in the same direction. If a grid like the one presented in Fig 15 is provided, it is possible to compute the number of lanes in each column by counting the number of subsets of adjacent cells moving in the same direction (the two examples provided should help understanding the concept). In the worst (or least organized) case the number of lanes correspond to the number of cells. Under perfect alignment a minimum of two lanes is formed (excluding unidirectional flow). It could be inferred (with a numerical validation given in S3 Appendix) that the expected value for the number of lanes is:

$$\begin{array}{c} \langle N_{lanes}\left(r,m\right)\rangle \;=2\cdot \left(1-m\right)\cdot r\cdot \left(r-1\right)+1 \end{array}$$ (12)

where *m* is the width of the “corridor” as indicated in Fig 15. Eq (12) is plotted in Fig 14(b) for different values of *m*. A quick comparison with the experimental results shows that Eq (12) allows to describe the average number of lanes in relation with the flow ratio. Obviously Eq (12) cannot describe all the cases, since it is an upper bound for the expected number of lanes in case of random motion. However, it is interesting to notice that the agreement between the theoretical expression and the experimental results is particularly good for case (c), which indeed represented a situation where self-organization was not possible (or very difficult).

In the theoretical analysis so far we have obtained an equation (Eq (9)) which suits well for the expressions of capacity showing a “U” shape and we were able to estimate the number of lanes from a theoretical perspective. The question remains on how to describe the “W” shape which often appeared in the literature. From a qualitative point of view we have seen in the experimental results that the formation of lanes contributes in increasing the organization and making the motion smoother. While it is very difficult to predict under which circumstances lanes form as this depends on a number of factors that are difficult to model (environment, presence of leaders, signage, familiarity with the location…), we can assume from a purely stochastic point of view that lanes are easier to form when the expected value is higher. For instance, creating 2 lanes in an hypothetical corridor having *m* = 5 cells, will be easier in the balanced configuration, where the expected number of lanes is 3, compared with a configuration having *r* = 0.1 where the expected number of lanes is 1.72. We also know that when a bidirectional stream get organized in lanes, the capacity will become equal to the unidirectional case, since interactions are only occurring within pedestrians moving in the same direction.

The above considerations lead to the conclusion that the increase in capacity given by the formation of organized lanes must be proportional to the expected number of lanes. Since lanes are easier to form in the balanced case, the gain in capacity given by the creation of organized and stable lanes must be higher compared to unbalanced configurations. We can therefore derive a modified version of *q*_*tot*_ taking into account the formation of organized structures. This function can be obtained by summing up *q*_*tot*_ with a transient term derived from the general form for the number of lanes, leading to the following expression:

$$\begin{array}{c} q_{tot}\left(r,n,\tau \right)=q_{tot}\left(r,n\right)+k\cdot r\cdot \left(r-1\right)\cdot \tau \end{array}$$ (13)

where *τ* is a transient term and *k* is a scaling factor to be determined using empirical data. By setting the minimum at a flow ratio of $\frac{1}{2}$ and the maximum for the unidirectional cases (and leaving *τ* = 0), it can be obtained:

$$\begin{array}{c} k=4\cdot (q_{min}-q_{max}) \end{array}$$ (14)

**Fig 16:**
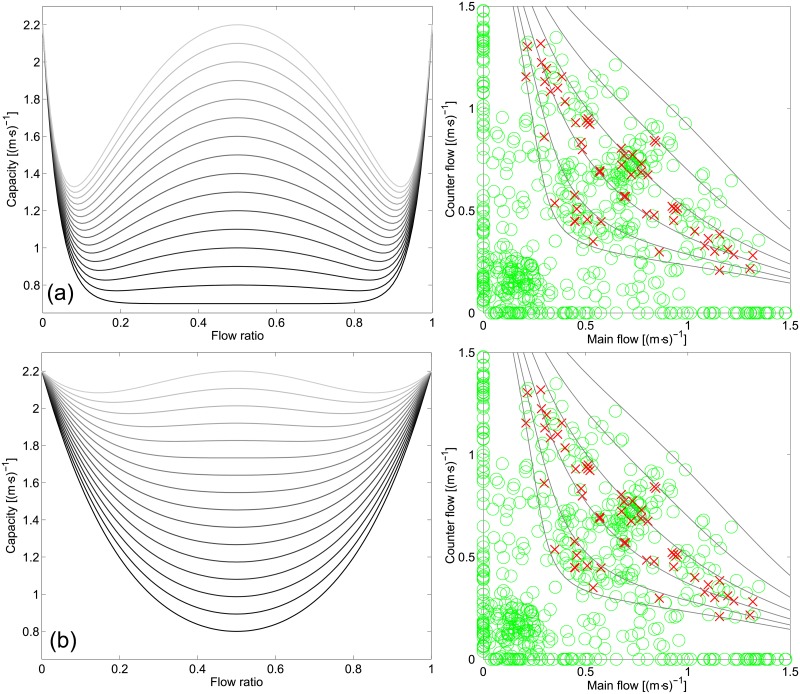
Transient capacity function and ability to depict transition to congestion. In the right side congested areas are given using red crosses, non-congested with green circles. In (a) a threshold of 1.00 m^−1^ for the relative rotation range is used to define congestion, in (b) the threshold is 3.00 m^−1^. Parameters used in the equations are: *n* = 25, *q*_*min*_ = 0.75 (m⋅s)^−1^ and *q*_*max*_ = 2.2 (m⋅s)^−1^ for (a) and *n* = 5, *q*_*min*_ = 0.80 (m⋅s)^−1^, *q*_*max*_ = 2.2 (m⋅s)^−1^. In the graphs on the left *τ* is varied from 0 to 1 (0 being the darkest line and 1 the lightest).

The left side of Fig 16 shows the unsteady form of *q*_*tot*_ plotted using typical values for *n*, *q*_*min*_ and *q*_*max*_ and letting the transient term *τ* vary from 0 to 1. *τ* contains all the different factors which could lead to the formation of organized lanes. In general, we can say that this process requires time (hence the name *τ*), but other factors such as training or signage (keep left/right or guided patterns) may contribute in increasing the value of *τ* and therefore improving the efficiency of the balanced flow. In other words, *τ* can be seen as a factor determining the degree of coordination among pedestrians; the higher *τ* it gets, the easier will be the formation of lanes and the higher will be the capacity of the balanced configuration.

To check the validity of the proposed function in describing the transient capacity of bidirectional streams we may use the relative rotation range to define a threshold for congestion. The graphs on the right side of Fig 16 show a categorization using two different thresholds of the relative rotation range based on the experiments with small group interactions (case (a)). A threshold of 1.00 m^−1^ is used in Fig 16(a) and 3.00 m^−1^ in Fig 16(b). Case (a) generally showed an uncongested motion and since the experiment represented a dynamic process it is not possible to fix a unique value for capacity. It is however seen that Eq (13) allows to define a minimum level for congestion in both cases, thus allowing a variable definition of capacity which takes into account the contribution of lanes.

From Fig 16, it is also possible to see that the function proposed here allows to reproduce most of the equations reported in the literature (see also S1 Appendix for details). In fact, by varying *τ* the shape of the function changes from the “U” (*τ* = 0) which was associated with short-term disorganized interactions to the “W” (*τ* = 1) which was associated with a semi-organized motion in lanes. Understanding under which conditions the transient term start playing an important role and how to predict if the flow will result into an organized form around the balanced configuration could be an interesting topic for future research. Also, a more complete definition for capacity could list *q*_*min*_, *n* and *τ* for different scenarios, thus allowing to define capacity for bidirectional streams with better precision and allow a more accurate design of pedestrian facilities. This would also allow a more flexible and systematic definition of levels of service.

## Bidirectional flow fundamental diagram

**Fig 17:**
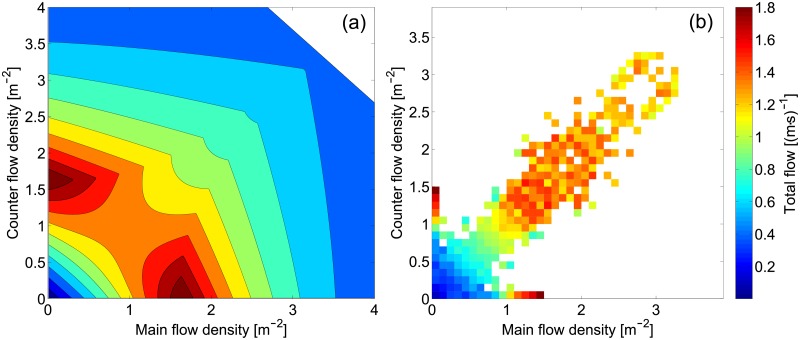
Bidirectional flow fundamental diagram. Comparison between experimentally obtained data and the particular FD by Flötteröd and Lämmel. The whole database is used to create the experimental fundamental diagram.

Finally, we want to conclude our discussion by comparing a particular form of the fundamental diagram, obtained by performing a complete analysis of our database, with the one provided by Flötteröd and Lämmel [54]. In their study, a theoretical (and three-dimensional) form of the FD for the bidirectional flow was derived. The authors used experimental data (roughly corresponding to our dataset E) to obtain the parameters defined in their functions. Fig 17 presents both the FD by Flötteröd and Lämmel and the one resulting from our database.

Although the number of points not covered by the analysis discussed here is rather large, it can be noted that, in general, the FD by Flötteröd and Lämmel reproduce well the characteristics of bidirectional flows. In particular, the maximum flow for the unidirectional case is correctly predicted being at about 1.5 m^−2^ and the change in flow along the symmetry line is also well depicted until the same density (1.5 m^−2^). However, when the density grows above 1.5 m^−2^, Flötteröd and Lämmel predict that over the symmetry line a somewhat linear drop in flow should occur, but this behavior is not observed in the FD resulting from our database. This can be related to the fact that Flötteröd and Lämmel used a limited number of experimental data to calibrate their model and therefore it becomes specific of the dataset used. Since, in general, qualitative features seem to be well described in their model, on-purpose experimental data could help increasing the reliability of their function.

## Conclusions

In this paper, we presented a throughout analysis on the characteristics of bidirectional pedestrian streams, finally leading to the definition of an equation for capacity which takes into account transient effects related to lane formation.

By comparing three cases, one where lane formation is hindered, one where lanes form easily and another one where lanes are quickly formed and dispersed, we have seen that when lanes are formed the bidirectional motion is split into a number of unidirectional streams moving in an orderly manner thus greatly improving capacity. In this regard, we have also confirmed that, as previous research speculated, the capacity of the balanced configuration is lower only when lanes are not formed, but quickly grows to eventually become equal to that of unidirectional streams once organized lanes get a stable structure.

A practical consequence of the above facts is that the balanced case is the one which requires the biggest attention, since the capacity to move people in both directions strongly depends on the self-organization of the crowd itself, which may be very difficult to influence, especially in critical situations. These considerations translate into two main requirements in regard to design and management of pedestrian facilities. On one side, they highlight the necessity to provide sufficient (but not redundant) guidance in pedestrian facilities, thus allowing a natural and smooth formation of lanes under changing conditions. On the other side, they draw attention to the risk existing when dense crowds need to move in opposite directions. In those cases, even the best practice in guidance and crowd control may not be sufficient and a drastic fall in capacity occurs when those measures fail. In brief, flow separation is an important requirement for scenarios where emergencies may happen.

From a theoretical aspect, the function for capacity proposed here allows to describe a variety of works presented in the literature and define a framework for categorizing bidirectional streams in a more systematic and accurate way. Although an absolute form defining values and parameters to be used is not provided, we defined methods and practices which can help to create more accurate definitions to be used in the Level of Service. For example, the framework presented here may help defining the efficiency of different signage strategies by measuring the *τ* values reached in different contexts. Also, long term observations of real situations may help defining a maximum level for congestion, which has the potential of becoming a universal criterion for determining capacity.

With this said, our study also suggested that it is not yet possible to summarize pedestrian flows into universal criteria determining human motion, since environmental and cognitive processes largely determine the outcome of pedestrian behavior. Relationship between people and the environment cannot be only based on geometrical features and, although we may be able to measure the degree of congestion, the reasons leading to it need to be considered on a case-by-case basis. However, for the specific case of the bidirectional flow, which represents a combination of stochastic (as our model showed) and cognitive aspects, it could be possible to summarize common environmental/cognitive elements and their contribution to capacity in a more systematic way, thus making the design of pedestrian facilities more accurate and reliable.

## Supporting information

S1 Data Set Pedestrian trajectories for the experiments considered in this work. Trajectories are divided into folders for the different experiments within the ZIP archive. Data relative to the own experiments are directly provided as a CSV file. Links are provided for data obtained from open database from third party researchers.
(ZIP)

S1 Fig Effect of flow ratio (literature review). Effect of the counter flow on bidirectional flow capacity. Geometries (crosswalk, sidewalk, corridor…) considered vary from author to author.
(EPS)

S2 Fig Experimental configurations. Test section (central corridor) and waiting (starting) areas for different types of experiment.
(EPS)

S3 Fig Example of randomly generated grids used to validate the different statistical equations relating to the effect of flow ratio. (a) represents a unidimensional case with 5 cell and (b) with 8 cells; (c) represents a bidimensional case with a length of 5 cells and an height of 3 cells.
(EPS)

S4 Fig Graphical representation of the error between empirical expressions and numerical results. In all the cases a clear convergence is seen as the number of iterations increases.
(EPS)

S1 Appendix Literature on bidirectional flow. This appendix contains a comprehensive overview on the literature on bidirectional pedestrian flow.
(PDF)

S2 Appendix Details on considered experimental datasets. This appendix contains details on experimental conditions and experimental setup for the datasets considered in the main manuscript.
(PDF)

S3 Appendix Monte Carlo validation of probabilistic equations. This appendix contains a numerical validation based on the Monte Carlo method for the statistical equations provided in the main manuscript.
(PDF)
