# Correction: A universal function for capacity of bidirectional pedestrian streams: Filling the gaps in the literature

**DOI:** 10.1371/journal.pone.0216314

**Published:** 2019-04-25

**Authors:** Claudio Feliciani, Hisashi Murakami, Katsuhiro Nishinari

In Fig 16, the graphs appearing on the right side of the figure have mistakenly been duplicated. Please see the correct Fig 16 here.

**Fig 16:**
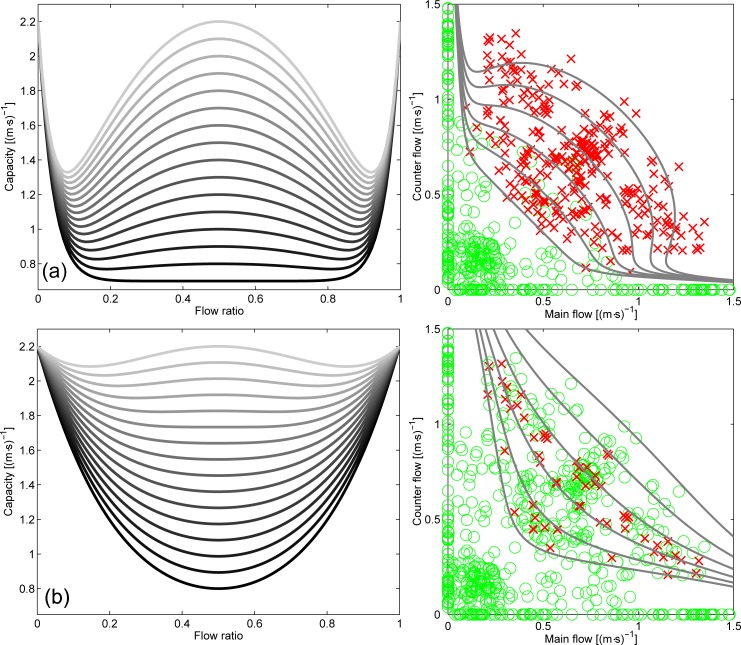
Transient capacity function and ability to depict transition to congestion. In the right side congested areas are given using red crosses, non-congested with green circles. In (a) a threshold of 1.00 m^−1^ for the relative rotation range is used to define congestion, in (b) the threshold is 3.00 m^−1^. Parameters used in the equations are: *n* = 25, *q*_*min*_ = 0.75 (m⋅s)^−1^ and *q*_*max*_ = 2.2 (m⋅s)^−1^ for (a) and *n* = 5, *q*_*min*_ = 0.80 (m⋅s)^−1^, *q*_*max*_ = 2.2 (m⋅s)^−1^. In the graphs on the left *τ* is varied from 0 to 1 (0 being the darkest line and 1 the lightest).
