# Supplementary material for: A universal function for capacity of bidirectional pedestrian streams: Filling the gaps in the literature
Source: PLoS One. 2018 Dec 19;13(12):e0208496. doi: 10.1371/journal.pone.0208496 (PMC6300270; doi:10.1371/journal.pone.0208496)
Supplement: S1 Appendix — This appendix contains a comprehensive overview on the literature on bidirectional pedestrian flow. (PDF) [file pone.0208496.s006.pdf]

S1 Appendix: Literature on bidirectional flow

This appendix contains more details on the works considered in the literature review. Readers interested in details regarding the context of each study and the equations presented from the different authors can find the relevant information in this section. This appendix is organized considering in historical order the studies that are the most relevant for the topics presented in this work. Readers interested in even further details are addressed to the relevant works by each author.

Navin and Wheeler

The first efforts to quantify the effects of the counter flow were reported by Navin and Wheeler [1]. In their study, the authors noted that when a small number of pedestrians was walking in the minor flow, they occupied a proportionally larger size of the walkway compared to the major flow. As the flow became balanced (close to a flow ratio of 0.5), both groups tended to form organized lanes equally taking half of the walkway. Weidmann [2] quantified this effect in terms of capacity reduction as illustrated in S1 Fig (part a).

Cheung

In the digital era, data acquisition became easier and Cheung was able to collect a large database of pedestrian speed and flow at different pedestrian facilities in Hong Kong [3]. The effect of counter flow was quantified in a number of situations, including stairways in both directions (up and down), escalators and different types of walkways ("passageways" in his work). For the case of flat walkways (which had different widths), Cheung observed (similarly to Navin and Wheeler) that in the presence of counter flow the opposing traffic contributes in reducing the total capacity, but when the flow becomes balanced, pedestrians in each direction share the width of the walkway equally. Cheung was able to provide an empirical function for the relation between capacity and flow ratio (the function provided here is obtained by combining the relative reduction in capacity and the unidirectional capacity provided by Cheung [3]):

$$q_{tot}(r) = q_{uni}\left[1 - (a_0 + a_1 r + a_2 r^2 + a_3 r^3 + +a_4 r^4 + +a_5 r^5 + a_6 r^6)\right] \tag{1}$$

where q_{uni} is the unidirectional capacity (given as 1.53 $(\mathrm{m{\cdot}s})^{-1}$) and a_i for $i = [0, 6]$ are empirically obtained parameters. It is important to remark that the function by Cheung is defined for $0 < r < 1$, which means that it is not continuous for the transition from unidirectional to bidirectional flow as shown in S1 Fig (part b). In the case of Cheung, the stability around the balanced flow is remarkable and practically the same capacity of unidirectional flow is obtained.

Lam et al.

Some years later Lam et al. used the same approach to investigate the case of crosswalks (always in Hong Kong), creating a large database including speed and flow for two different locations (a commercial area and a shopping facility) [4]. Facilities considered by Lam et al. had a very large width (7.2 m and 9.0 m respectively). Based on their observations they derived an empirical function for the effective flow (defined as the total flow relative to each direction), given by:

$$q_{eff}(r) = b_0 + b_1 r + b_2 r^2 + b_3 r^3 \tag{2}$$

where r is the flow ratio and b_0, b_1, b_2 and b_3 are experimentally obtained parameters. To obtain the bidirectional flow capacity (the sum of flows in both directions) the following transformation is required:

$$q_{tot}(r) = r \cdot q_{eff}(r) + (1-r) \cdot q_{eff}(1-r) \tag{3}$$

$$= c_0 + c_1 r + c_2 r^2 + c_3 r^3 + c_4 r^4 \tag{4}$$

with c_0, c_1, c_2, c_3 and c_4 given by:

$$c_0 = b_0 + b_1 + b_2 + b_3 \tag{5}$$

$$c_1 = -2b_1 - 3b_2 - 4b_3 \tag{6}$$

$$c_2 = 2b_1 + 3b_2 + 6b_3 \tag{7}$$

$$c_3 = -4b_3 \tag{8}$$

$$c_4 = 2b_3 \tag{9}$$

Using the empirical parameters provided by Lam et al. it is possible to plot the relation between capacity and flow ratio as shown in S1 Fig (part *c*). Qualitatively the result is very similar to the previous studies although the advantage gained when the flow becomes balanced is less marked.

In regard to the study by Lam et al. it should be remarked that the interpretation of the function proposed by the author is rather controversial and there is no agreement between researchers (Flötteröd [11] who also based part of his research on the conclusions by Lam et al. expressed doubts on the interpretation of the results). In addition, the author did not provide an official explanation and the presentation is rather unclear. As a consequence, the transformation proposed above is also based on different qualitative remarks made in his work.

Kretz et al.

To study more in detail phenomena related with bidirectional flows, Kretz et al. [5] performed a supervised experiment in a corridor (slightly less than 2 m in width). Two groups of pedestrians (67 participants in total) waited in designated areas located 20 m from each other's inside the corridor. After the start signal was given, both groups walked toward each other's crossing in the middle of the corridor. Video recordings were taken at three different positions and were manually analyzed. The results of their study on counter flow effects are given in S1 Fig (part *d*) ("start", "center" and "end" refer to the three relative positions inside the corridor). In contrast to the previous researchers, they found that bidirectional flow performs better than unidirectional one in any situation (the study by Kretz et al. did not specifically consider capacity as the main goal, but such values can be obtained from their results). This conclusion may be partially related to the very narrow corridor used (less than 2 m, allowing to form typically 2 or 3 lanes) and the relative small group size considered (especially when compared with the field studies presented earlier where thousands of people were observed). These particular conditions may also explain the remarkable capacity obtained by Kretz et al. exceeding 3 $(\text{m}\cdot\text{s})^{-1}$, which is the highest among the different studies considered here. As a consequence, the study by Kretz et al. represents a particular (but yet significant) case among the ones considered here.

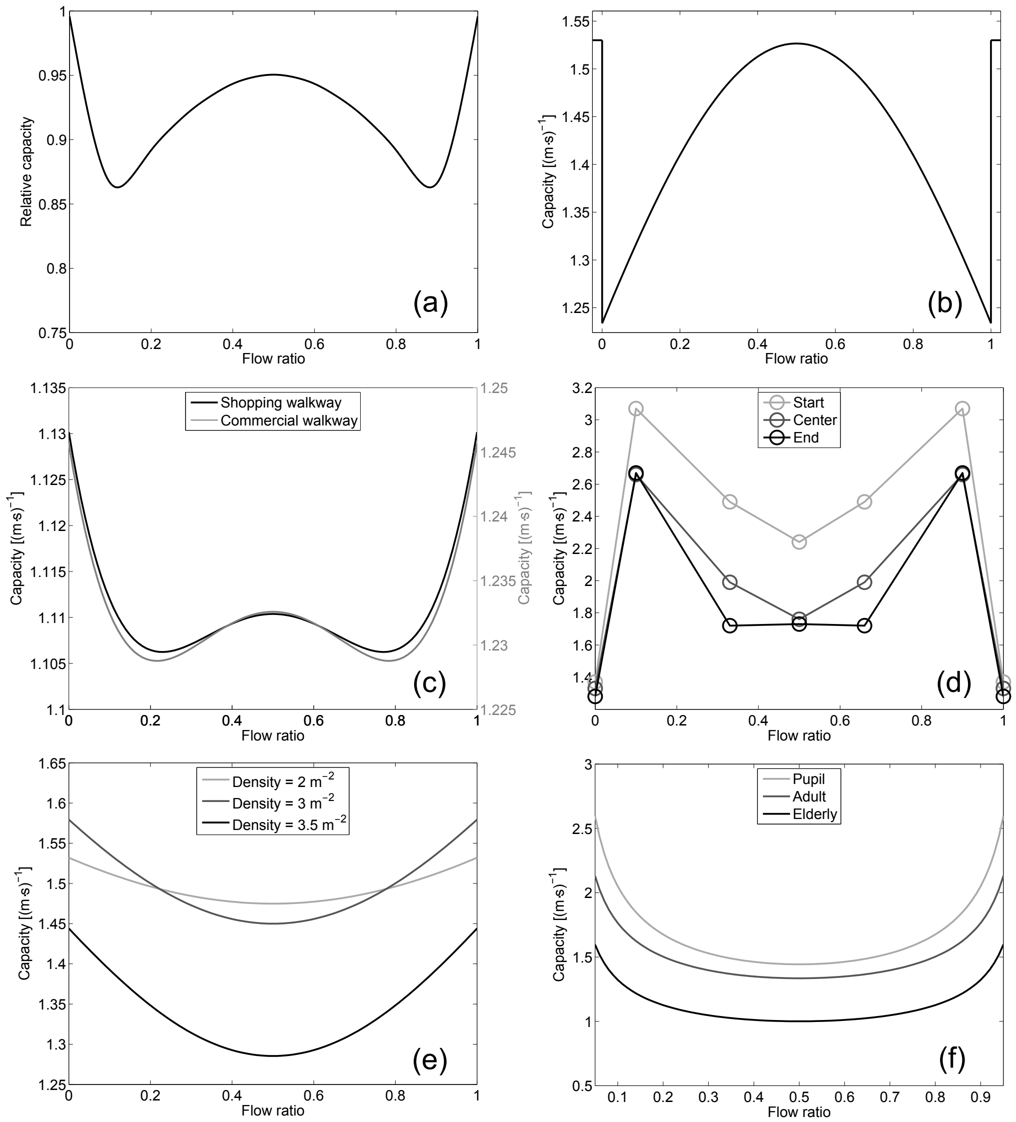

S1 Fig. Effect of flow ratio (literature review). Effect of the counter flow on bidirectional flow capacity. Geometries (crosswalk, sidewalk, corridor...) considered vary from author to author.

Wong et al.

Wong et al. [6] have studied different configurations of cross-flows in a supervised experiment, with the bidirectional (counter) flow considered as the most extreme case of the ones studied. 90 participants were recruited for the experiment and experimental procedures were similar to the ones of Kretz et al. (i.e. two groups of pedestrians waiting in separate areas before starting). The section considered was 3 m in width. Wong et al. developed an algorithm to extract pedestrians' position at each video frame, which allowed them to work with a large number of accurate data. The database was fitted with a function they proposed to predict pedestrian group velocity based on density ρ_{tot} and intersecting angle ϕ. With v_{free} being the free walking speed, their function is given by:

$$v_{mono}(\rho_{tot}, r, \phi) = v_{free} \exp\left[-\theta_1 \rho_{tot}^2\right] \cdot \exp\left[-\theta_2 (1 - \cos\phi) \left((1-r)\rho_{tot}\right)^2\right] \quad (10)$$

with θ_1 and θ_2 being experimentally determined parameters. The bidirectional counter flow case is obtained by setting $\phi = 180°$, which leads to the velocity for each monodirectional group:

$$v_{mono}(\rho_{tot}, r) = v_{free} \exp\left[-\theta_1 \rho_{tot}^2\right] \cdot \exp\left[-2 \cdot \theta_2 \left((1-r)\rho_{tot}\right)^2\right] \quad (11)$$

which can be promptly converted into pedestrian flow by multiplying with the corresponding density and flow ratio as follows:

$$q_{mono}(\rho_{tot}, r) = v_{mono}(\rho_{tot}, r) \cdot \rho_{tot} \cdot r \quad (12)$$

Finally, the capacity can be computed by summing up both monodirectional components:

$$q_{tot}(\rho_{tot}, r) = q_{mono}(\rho_{tot}, r) + q_{mono}(\rho_{tot}, 1-r) \quad (13)$$

S1 Fig (part e) shows the behavior of the above function for different densities by using the numerical values provided by Wong et al. ($v_{free} = 1.034$ m/s, $\theta_1 = 0.075$ m^4 and $\theta_2 = 0.019$ m^4). In the central section (around 0.5) a behavior similar to the work by Kretz et al. is observed, but the function is continuous at the transition between uni- and bidirectional flow. Wong et al. predict that bidirectional flow is always performing worse than unidirectional one.

Alhajyaseen et al.

Alhajyaseen et al. [7, 8] developed what they defined as a drag force model based on empirical observations of signalized crosswalks of different dimensions (all were larger than 4 m in width), from which they were able to gain trajectories of crossing pedestrians. Their capacity function for the flow in one direction takes the following form:

$$q_{mono}(r) = d_0 r^{d_1} (1-r)^{d_2} \quad (14)$$

where d_0, d_1 and d_2 are experimentally determined parameters. Again, we can obtain the function for the total flow by summing up both monodirectional components as follows:

$$q_{tot}(r) = q_{mono}(r) + q_{mono}(1-r) = d_0 \cdot \left[r^{d_1}(1-r)^{d_2} + (1-r)^{d_1} r^{d_2}\right] \quad (15)$$

Alhajyaseen et al. obtained different parameters for three age groups: pupils, adults and elderly. Their capacity for the three age groups is given in S1 Fig (part f). Their function shows similar properties with the ones previously presented: numerical values are close to the ones by Kretz et al. and function shape resembles to the one proposed by Wong et al. However, the function by Alhajyaseen et al. tends to infinity for unidirectional configurations, thus not allowing a continuous transition to the bidirectional case.

Feliciani and Nishinari

In our previous research, a bidirectional flow moving inside a subway station has been studied by analyzing video recordings obtained from multiple cameras [9]. Based on the difference between the (in)flow of pedestrians entering a test section and the (out)flow of the ones leaving it, we were able to make qualitative and quantitative distinctions on the different flow regimes and make a rough estimation of capacity. However, we were not able to determine with sufficient accuracy the relationship between flow ratio and capacity and we simply concluded that a non-linear relationship is expected and balanced case should have the lowest value.

Zhang et al.

Very recently, Zhang et al. [10] performed a controlled experiment for the case of crosswalks. Conditions similar to reality were recreated in laboratory, with pedestrians waiting at opposite sides of a section which was later crossed by both groups simultaneously (a setup similar to the one of Kretz et al. and Wong et al.). Investigated crosswalk had a length of 7.5 m and a width of 4.0 m. Number of pedestrians on both sides was changed to study flow ratio, with a maximum of 100 pedestrians in total. Similar to some previous studies, Zhang et al. concluded that capacity in one direction is given by a cubic function:

$$q_{mono}(r) = e_0 r^3 + e_1 r^2 + e_2 r \tag{16}$$

where $e_0 = 8.7$ (m·s)$^{-1}$, $e_1 = -12.4$ (m·s)$^{-1}$ and $e_2 = 5.9$ (m·s)$^{-1}$ are empirical values. As usual the total capacity is obtained by summing main and counter flow, resulting in:

$$q_{tot}(r) = q_{mono}(r) + q_{mono}(1-r) = (2e_1 - 3e_0)r^2 - (2e_1 + 3e_0)r + (e_0 + e_1 + e_2) \tag{17}$$

which is a simple quadratic equation. Based on their results, Zhang et al. concluded that the capacity of bidirectional flow in crosswalks is close the one for unidirectional flow in corridors. Concerning lane formation, Zhang et al. interestingly noted that it neither depends on the number of pedestrians in bidirectional flow nor on the flow ratio.

References

1. Navin FP, Wheeler RJ. Pedestrian flow characteristics. Traffic Engineering, Inst Traffic Engr. 1969;39.

2. Weidmann U. Transporttechnik der Fussgänger: Transporttechnische Eigenschaften des Fussgängerverkehrs (Literaturauswertung). ETH, IVT; 1993. doi:10.3929/ethz-a-000687810.

3. Cheung Cy. Pedestrian flow characteristics in the Hong Kong Mass Transit Railway stations. The Hong Kong Polytechnic University; 1998. Available from: http://hdl.handle.net/10397/3447.

4. Lam WH, Lee JY, Cheung C. A study of the bi-directional pedestrian flow characteristics at Hong Kong signalized crosswalk facilities. Transportation. 2002;29(2):169–192. doi:10.1023/A:1014226416702.

5. Kretz T, Grünebohm A, Kaufman M, Mazur F, Schreckenberg M. Experimental study of pedestrian counterflow in a corridor. Journal of Statistical Mechanics: Theory and Experiment. 2006;2006(10):P10001. doi:10.1088/1742-5468/2006/10/P10001.

6. Wong S, Leung W, Chan S, Lam WH, Yung NH, Liu C, et al. Bidirectional pedestrian stream model with oblique intersecting angle. Journal of transportation Engineering. 2010;136(3):234–242. doi:10.1061/(ASCE)TE.1943-5436.0000086.

7. Alhajyaseen WK, Nakamura H. Quality of pedestrian flow and crosswalk width at signalized intersections. IATSS research. 2010;34(1):35–41. doi:10.1016/j.iatssr.2010.06.002.

8. Alhajyaseen WK, Nakamura H, Asano M. Effects of bi-directional pedestrian flow characteristics upon the capacity of signalized crosswalks. Procedia-Social and Behavioral Sciences. 2011;16:526–535. doi:10.1016/j.sbspro.2011.04.473.

9. Feliciani C, Nishinari K. Phenomenological description of deadlock formation in pedestrian bidirectional flow based on empirical observation. Journal of Statistical Mechanics: Theory and Experiment. 2015;2015(10):P10003. doi:10.1088/1742-5468/2015/10/P10003.

10. Zhang J, Cao S, Seyfried A. Properties of Pedestrian Movement at Signalized Crosswalk. In: Proceedings of Pedestrian and Evacuation Dynamics 2016. University of Science and Technology of China Press; 2016. p. 126–130.

11. Flötteröd G, Lämmel G. Bidirectional pedestrian fundamental diagram. Transportation research part B: methodological. 2015;71:194–212. doi:10.1016/j.trb.2014.11.001.
