# Supplementary material for: A universal function for capacity of bidirectional pedestrian streams: Filling the gaps in the literature
Source: PLoS One. 2018 Dec 19;13(12):e0208496. doi: 10.1371/journal.pone.0208496 (PMC6300270; doi:10.1371/journal.pone.0208496)
Supplement: S2 Appendix — This appendix contains details on experimental conditions and experimental setup for the datasets considered in the main manuscript. (PDF) [file pone.0208496.s007.pdf]

# S2 Appendix: Details on considered experimental datasets

This appendix provides details on the datasets used to investigate bidirectional streams throughout this work. Readers interested in the reasoning behind the 3-group division made up to analyze the data should find the answers while reading this section. Readers interested in small details and trying to understand subtle differences among the results presented for the 3 types of bidirectional flow can also find some clues by checking this appendix. For further details, readers are advised to verify the content of each study by directly referring to the work of each author (references are provided in S1 Table).

## Technical details of the experimental data

S1 Table and S2 Table present some of the characteristics of the experiments included in our database.

**S1 Table. Experiments considered in the empirical database. In providing the total time for each dataset we are referring to that portion of the experiments which has been used in data analysis (therefore excluding breaks and dead times).**

| # | Authors | Reference | Sensor | Participants | Runs | Total time |
|---|---|---|---|---|---|---|
| A | Feliciani and Nishinari | [1] | Single camera | 54 | 16 | 12 min |
| B | Feliciani and Nishinari | [2] | Single camera | 50 | 11 | 8.5 min |
| C | Gorrini et al. | [3, 4] | Single camera | 54 | 21 | 13 min |
| D & E | Zhang et al. | [5–8] | 2 cameras | ≈ 300 | 22 | 25 min |

Data for experimental datasets A–C are directly given in the supporting information S1 Data Set. Data for datasets D and E are openly available online and URLs are provided as comments within the supporting information S1 Data Set. In the experiments by Zhang et al. (D & E) images from two different cameras were combined to cover the full length of the corridor.

**S2 Table. Scenario dimensions, crowd properties and social structure for the considered cases. "Side/destination" refers to the condition if pedestrians were aiming to a particular side of the corridor (left/right) or just intended to cross it (side/destination irrelevant).**

| | | | Corridor size | | Maximum | | |
|---|---|---|---|---|---|---|---|
| # | Side/destination | Social group | Length | Width | Density | Flow | Flow ratio |
| A | Free choice | Individuals | 10 m | 3.0 m | 1.5 m$^{-2}$ | 2 (m·s)$^{-1}$ | 0, 0.17, 0.33, 0.50 |
| B | Free choice | Individuals | 10 m | 2.4 m | 1.5 m$^{-2}$ | 2 (m·s)$^{-1}$ | 0, 0.25, 0.50 |
| C | Free choice | Mixed | 10 m | 3.0 m | 1.7 m$^{-2}$ | 2 (m·s)$^{-1}$ | 0, 0.17, 0.33, 0.50 |
| D | Free choice | Individuals | 8 m | 3.6 m | 2 m$^{-2}$ | 1.7 (m·s)$^{-1}$ | 0.4, 0.5 |
| E | Fixed | Individuals | 8 m | 3.0 m & 3.6 m | 3 m$^{-2}$ | 1.8 (m·s)$^{-1}$ | 0.5 |

Many studies specifically considered the balanced bidirectional flow, so most of the data regard cases with a flow ratio close to 0.5. The number of pedestrians involved in each experimental campaign varies greatly from study to study, with the lowest figure being of 50 participants and the highest slightly over 300. Most of the studies focused on pedestrians behaving individually, with the exception of the study in C, which specifically considered a given number of pairs among the participants (about 40% behaved in pairs).

As a whole, the database created allows to study the dynamics of bidirectional flow from a density of about 0.1 $m^{-2}$ up to a maximum of 3 $m^{-2}$. Pedestrian flow also covers the range of values typically reported in the literature, with a maximum value of about 2 $(m{\cdot}s)^{-1}$. Geometrical dimensions of the corridors were quite similar (width changes from a minimum of 2.4 m up to a maximum of 3.6 m), which represent an advantage for comparisons but may limit the universality in the conclusions.

One of the most distinguishing element between the different cases concerns the destination choice. For the cases A–D participants to the experiments were able to choose freely the side from which leave the corridor after traversing it (either right, left or straight). Under this condition, lanes can easily form and tend to be stable. In those cases, pedestrians were simply asked to walk toward the exit (on the opposite side of the corridor) without any specific order.

In the particular case of dataset E, half of the participants were asked to leave the corridor from the left side and the other half from the right side (thus creating a sort of cross flow inside the corridor). In this case, pedestrians were therefore aiming to a particular destination and had to cross the corridor in an oblique direction.

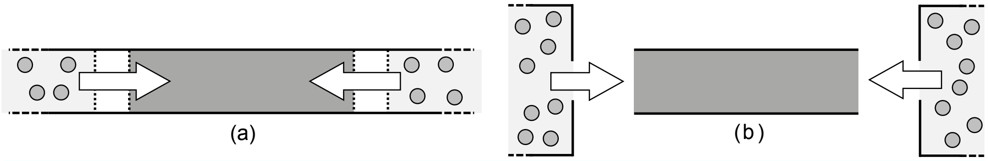


**S2 Fig. Experimental configurations.** Test section (central corridor) and waiting (starting) areas for different types of experiment.

It is also important to briefly mention that geometries were not the same for all the experiments and different strategies were used to calibrate the flow ratio when needed. In A–C two opposite located waiting areas with equal surface were created as shown on both sides of S2 Fig (part *a*). Participants were uniformly distributed inside each waiting area with the number being changed for different configurations. The flow ratio was therefore changed by changing the density of both groups and keeping the overall density constant. Zhang et al. (D & E) used a different strategy: two waiting areas with a small exit were created at both sides of the corridor studied as shown in S2 Fig (part *b*). By changing the width of the opening on each side, the flow for each direction was also changed, thus allowing them to have a control on it and also influencing the flow ratio.

Finally, we also have to acknowledge some limitations on the experimental conditions represented in our database. In particular, we are aware that heterogeneity is considered only in a minimal extent and the same can be said in regard to social structures. Campanella et al. [9] clearly showed that desired speed, body size and reaction time can affect flow efficiency and Gorrini et al. [10] arrived to similar conclusions in relation to grouping patterns. Since the studies considered here mostly deal with adult pedestrians (mostly young people) and grouping with no more than two persons, further investigations would have to be done to determine the impact when a large population is composed, for example, by elderlies or pupils (although Alhajyaseen et al. only found minor quantitative differences in this regard).

## Grouping criteria and underlying reasoning

Based on the discussion provided in the main text and the specific characteristics of each experiment given above, the different datasets were combined into three categories as shown in S3 Table.

**S3 Table. Grouping of datasets with similar properties.**

| Group | Case study | Dataset(s) |
|---|---|---|
| (a) | Natural small group interaction | A & B & C |
| (b) | Facilitated lane formation (learning process) | D |
| (c) | Strong obstruction with forced motion | E |

The categories reported in S3 Table are not relative to geometrical, traffic (flow, density and speed) or social properties, but they rather refer to the methodological procedure used in the experiments.

Experiments in group (a) considered a small number of participants instructed to walk in a natural way in a mock corridor. Interactions were observed for a short time, since both groups rapidly passed through each other's. Also, because of the geometrical setup of the experiments (a long corridor in which participants need to walk "straight" for a long time) and the re-shuffling of participants performed by the staff in the start area, each case may be considered separately (i.e. it is very unlikely that participants developed a preferential strategy in crossing the corridor by executing the different experiments).

This was not the case in group (b), since participants rapidly learned that taking half of the corridor for each direction is the optimal solution. In fact, during the first execution at low densities it is seen that participants hesitate on which side of the corridor take after exiting the door in the waiting area. However, partially thanks to the low density, participants rapidly understood that taking each half of the corridor was beneficial in reducing the number of collisions. Since experiments were performed in Germany, which is a right-driving country, the choice to take each right half of the corridor came as a natural instinct. After exiting from the right side, participants returned in the waiting room. For the ones having performed the experiment already, it was clear that directly moving to the right side upon entering the corridor was the best solution to avoid unwanted collisions.

However, in the experiments of group (c), although the number of participants and the geometrical setup was identical to the experiments in group (b), the particular instructions contributed in creating a very different outcome. In fact, because of the instructions provided (half of the people were asked to exit from the left side and half from the right), people were not able to get organized and lanes were not observed if not locally for a short time. The experiments of group (c) needs therefore to be considered separately as the instructions given to participants were specifically designed to have an unnatural behavior where people are not able to act as they would in a normal condition. In some way, (a) and (b) are similar in terms of the instructions given, but they differ in terms of number of participants, geometrical setup and the possibility to learn (and "get better" during the experimental campaign). Group (c) mostly differs in terms of experimental procedure.

Given the above discussion, we judged reasonable considering the 3 categories separately and investigating what are the differences among them in regard to the concepts of lane formation and phase transition which are the main topics of this work.

## References

1. Feliciani C, Nishinari K. Empirical analysis of the lane formation process in bidirectional pedestrian flow. Phys Rev E. 2016;94:032304. doi:10.1103/PhysRevE.94.032304.

2. Feliciani C, Nishinari K. Pedestrians rotation measurement in bidirectional streams. arXiv preprint arXiv:161007185. 2016;.

3. Gorrini A, Crociani L, Feliciani C, Zhao P, Nishinari K, Bandini S. Social Groups and Pedestrian Crowds: Experiment on Dyads in a Counter Flow Scenario. arXiv preprint arXiv:161008325. 2016;.

4. Crociani L, Gorrini A, Feliciani C, Vizzari G, Nishinari K, Bandini S. Micro and macro pedestrian dynamics in counterflow: the impact of social groups. arXiv preprint arXiv:171108225. 2017;.

5. Zhang J, Klingsch W, Schadschneider A, Seyfried A. Ordering in bidirectional pedestrian flows and its influence on the fundamental diagram. Journal of Statistical Mechanics: Theory and Experiment. 2012;2012(02):P02002. doi:10.1088/1742-5468/2012/02/P02002.

6. Zhang J. Pedestrian fundamental diagrams: Comparative analysis of experiments in different geometries. Universität Wuppertal; 2012. Available from: `http://hdl.handle.net/2128/4898`.

7. Saberi M, Mahmassani H. Exploring areawide dynamics of pedestrian crowds: three-dimensional approach. Transportation Research Record: Journal of the Transportation Research Board. 2014;2421(1):31–40. doi:10.3141/2421-04.

8. Saberi M, Aghabayk K, Sobhani A. Spatial fluctuations of pedestrian velocities in bidirectional streams: Exploring the effects of self-organization. Physica A: Statistical Mechanics and its Applications. 2015;434:120–128. doi:10.1016/j.physa.2015.04.008.

9. Campanella M, Hoogendoorn S, Daamen W. Effects of heterogeneity on self-organized pedestrian flows. Transportation Research Record: Journal of the Transportation Research Board. 2009;2124:148–156. doi:10.3141/2124-14.

10. Gorrini A, Bandini S, Vizzari G. Empirical investigation on pedestrian crowd dynamics and grouping. In: Traffic and Granular Flow'13. Springer; 2015. p. 83–91. doi:10.1007/978-3-319-10629-8_10.
