# Supplementary material for: A universal function for capacity of bidirectional pedestrian streams: Filling the gaps in the literature
Source: PLoS One. 2018 Dec 19;13(12):e0208496. doi: 10.1371/journal.pone.0208496 (PMC6300270; doi:10.1371/journal.pone.0208496)
Supplement: S3 Appendix — This appendix contains a numerical validation based on the Monte Carlo method for the statistical equations provided in the main manuscript. (PDF) [file pone.0208496.s008.pdf]

S3 Appendix: Monte Carlo validation of probabilistic equations

The equations provided in the manuscript (repeated in S4 Table for convenience) have not been obtained empirically, but were derived analyzing results from simple computer simulations. Owning to the fact that statistical distributions have common elements in the equations describing them, we computed numerical results using a simple code and later tried to guess the equation following those data. Later, we systematically tested the empirical expression running a computer code with an increasingly larger number of iterations. While this is not a general proof, results presented below clearly showed that numerical results converge to analytical expressions as the number of iterations is increased.

S4 Table. Empirical expressions which have been numerically validated using a Monte Carlo method. Their derivation was based on a combination of theoretical and intuitive methods.

Equation name	Mathematical expression
Open-path probability	$p_{open\ path}(r,n) = (-1)^n \cdot [(r-1)^n + (-r)^n]$
Expected number of lanes	$\langle N_{lanes}(r,m)\rangle \ = 2 \cdot (1-m) \cdot r \cdot (r-1) + 1$
Expected order parameter	$\langle \Phi \rangle \ = 4\left(1-\frac{1}{n}\right) \cdot r \cdot (r-1) + 1$

In the specific, we wrote a computer code randomly creating the configurations of S3 Fig (relative to the equations provided in S4 Table) according to a given flow ratio. More in detail, each cell was assigned with a left direction when a random number was lower than flow ratio r and with the right direction instead (if the random number was bigger or equal than r). For the unidimensional case, this approach resulted in configurations similar to the examples presented in S3 Fig (part a) and S3 Fig (part b). We performed simulations by changing the flow ratio from 0 to 1 in 0.1 steps. The number of elements (cells) was changed from 1 to 10 in single steps for the unidimensional equations (open-path probability and number of lanes). In the case of the expected order parameter a bidimensional representation is required and therefore we changed both length and width from 1 to 5 cells in single steps (see S3 Fig, part c, for an example).

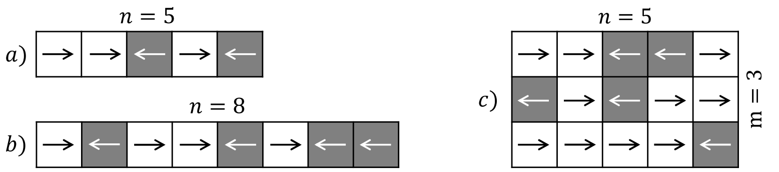

S3 Fig. Example of randomly generated grids used to validate the different statistical equations relating to the effect of flow ratio. (a) represents a unidimensional case with 5 cell and (b) with 8 cells; (c) represents a bidimensional case with a length of 5 cells and an height of 3 cells.

The number of tests (i.e. the number of combinations tested using random numbers) was varied from 10^3 to 10^7 by performing exponential steps (i.e. the exponent was

increased from 3 to 7). The error was computed taking that relative squared difference between the results obtained by simulation and the analytical result (obtained using the equation provided in the manuscript). A single error is taken by considering all combinations of flow ratio and grid size. Results for this Monte Carlo analysis are presented in S5 Table and S4 Fig.

S5 Table. Error between the exact solution and the numerical computation for different expressions introduced in the manuscript using a Monte Carlo method. Number refers to the equation number in the manuscript.

	**Iterations**				
Equation name	**1'000**	**10'000**	**100'0000**	**1'000'000**	**10'000'000**
Open-path probability	609.56%	13.312%	1.3514%	0.30697%	0.018119%
Expected number of lanes	1.5699%	0.14759%	0.013429%	0.0015203%	0.00011596%
Expected order parameter	6.6407%	0.59696%	0.075762%	0.0087961%	0.00066378%

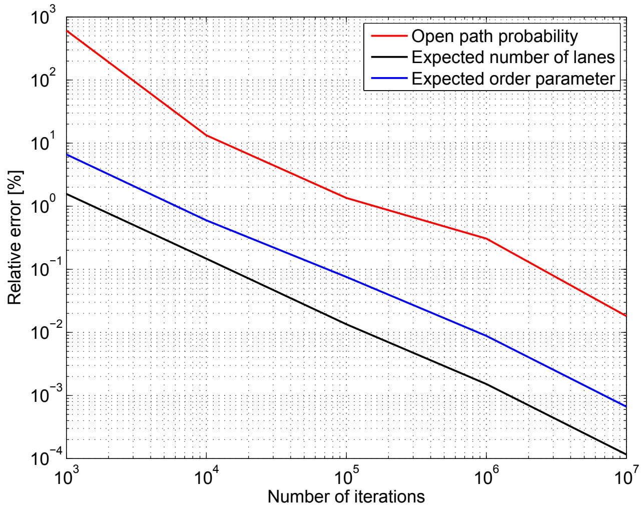

S4 Fig. Graphical representation of the error between empirical expressions and numerical results. In all the cases a clear convergence is seen as the number of iterations increases.

The three equations converge with the same speed in logarithmic terms, thus showing a quick convergence toward the analytical solution. Open-path probability has the highest error, but this is below 1% for one million iterations and rapidly fall below 0.1% for ten millions. Equations for the expected number of lanes and the order parameter already have small errors for low iterations and errors below 0.001% are reached for ten millions iterations. In general, it is clearly seen that in all the cases the numerical results converge to the analytical one when the number of iterations is increased (error gets close to zero), thus showing that equations provided in the manuscript are very likely to be correct (since this is not a general proof, we cannot affirm it in absolute term).
